# Understanding non-nutritive oral behaviors in dairy calves (*Bos taurus*): A systematic review protocol

**DOI:** 10.1371/journal.pone.0319778

**Published:** 2025-03-20

**Authors:** Christina R. Doelling, Sarah Kappel, Marina A. G. von Keyserlingk, Daniel M. Weary

**Affiliations:** Animal Welfare Program, Faculty of Land and Food Systems, The University of British Columbia, Vancouver, British Columbia, Canada; National Research Centre, EGYPT

## Abstract

This protocol outlines the proposed aims, rationale, study design, methods, and dissemination plan for a systematic review focusing on non-nutritive oral behaviors in dairy calves. The primary outcome measures of interest are the occurrence, frequency, and duration of these behaviors, with a specific focus on cross-sucking of pen mates. The review assesses how farm management interventions affect the occurrence, frequency, and duration of non-nutritive oral behaviors in dairy calves and informs recommendations regarding management practices that mitigate cross-sucking and other non-nutritive oral behaviors. Where applicable, PRISMA-P guidelines are followed, and all data will be made publicly available at the time of publication.

## Introduction

On many dairy farms, calves (*Bos taurus*) are separated from the dam hours after birth [1] and then reared individually or socially with other calves [2]. Young calves are highly motivated to suckle from their dam [3] but early separation from the cow prevents the calf from expressing this natural suckling behavior [1]. Under artificial rearing conditions, milk feeding varies in terms of the volume of milk provided (ranging on average from 10% to 20% of calf body weight [4,5]), the number of meals per day (e.g., once, twice, or multiple times throughout the day [6]), and the method of milk provision (e.g., via a bucket, teat bottle, or automatic milk feeder [7]). Under these conditions, calves will sometimes redirect sucking behavior to pen fixtures or pen mates; these oral behaviors not related to the ingestion of food can be collectively referred to as non-nutritive oral behaviors (NNOBs) [8,9]. Some NNOBs can persist after weaning, and sucking on other individuals (i.e., “cross-sucking”) may increase the risk of teat and other injuries [10] and facilitate disease transfer [11], potentially discouraging farmers from housing dairy calves socially.

A number of studies have considered the origin, prevalence, and management of NNOBs. Some of these findings were covered in a narrative review published more than two decades ago [12]. The authors of this review concluded that a myriad of factors affect NNOBs, such as feeding method, age, and weaning strategy. However, the evidence available at that time was insufficient to draw clear conclusions about the relationship between NNOBs and different management factors. Subsequent research findings are mixed. For instance, Nielsen and colleagues [13] found that employing a gradual weaning method, as opposed to abrupt, reduced cross-sucking rates in dairy calves. However, de Passillé and colleagues [14] found no relationship between weaning method and cross-sucking. These discrepancies in study findings highlight that cross-sucking and related management factors are still poorly understood.

To the best of our knowledge, there has been only one systematic review on NNOBs in cattle, and this study focused exclusively on the effect of diet (solid feed and roughage inclusion from birth through post-weaning/adulthood) and was not specific to dairy calf rearing [15]. The current review differs in that it specifically focuses on dairy calves and the effects of management factors in addition to diet. The primary objective of our systematic review is to synthesize research findings on NNOBs in dairy calves and summarize evidence relating to how management practices relate to the prevalence of cross-sucking and other NNOBs. Given that milk feeding method (i.e., bucket *versus* teat feeding) and weaning practices (i.e., abrupt *versus* gradual) are likely to affect motivation to suck [16], we hypothesize that these factors will be associated with NNOBs in dairy calves. This systematic review aims to identify the management factors that reduce NNOBs in dairy calves and thus inform recommended practices for dairy farms to lower the prevalence of these behaviors. To provide tailored management suggestions that will be beneficial to a wide range of different dairy farms, we plan to develop recommendations in consideration of different dairy cattle breeds and production scales (i.e., small-scale dairy farms and larger commercial herds). Finally, we aim to gain a better understanding of the etiology of NNOBs in calves, the mechanisms by which these behaviors are maintained over time, and the long-term effects of these behaviors on health, production, and welfare.

## Materials and methods

### Study design, eligibility, and selection criteria

We developed our review protocol following the Preferred Reporting Items for Systematic Reviews and Meta-Analyses Protocols (PRISMA-P) guidelines [17] (S1 Table). The first stage was to identify study aims as described above. The following stages included creating the eligibility criteria, searching for relevant articles, round one of screening of articles, round two screening of retained articles, data extraction, quality assessment, risk of bias assessment, data synthesis and summary.

Two independent reviewers (authors CRD and SK) created the eligibility criteria using the Population, Intervention, Comparison, and Outcome (PICO) framework [18] with input from DMW and MvK. Reasons for excluding a study were recorded in a PRISMA-P Flowchart (S2 Table). DMW and MvK were asked to resolve any questions about eligibility uncertainty. The inclusion and exclusion criteria, outlined in the PICO framework, are shown below in Table 1.

**Table 1:** Eligibility of peer reviewed articles focused on management practices that are associated with reduced non-nutritive oral behaviors in dairy calves, using the PICO framework.

PICO	Inclusion Criteria	Exclusion Criteria
Population	Research completed on a dairy farm Dairy breeds of *Bos taurus* and *Bos indicus* Dairy x beef if raised on dairy farm Calves from birth through weaning	Beef breeds Buffalo Primiparous and multiparous cows Non-dairy production types (e.g., veal or beef calves)
Intervention	Weaning methods Milk feeding frequency Enrichment exposure Group size Group composition Feeding method Milk allowance Milk flow rate Hay/ roughage provision Weaning duration Water provision Housing type Diet, feed supplementation Genetic/breed effects Human interaction	Anti-suck devices (e.g., nose clips) Surgical interventions
Comparison	Effect of intervention on NNOBs occurrence, frequency, and/or duration	No treatment or control group Observational only
Outcomes	Occurrence, frequency, and/or duration of NNOBs, including cross-sucking and other abnormal oral behaviors (e.g., tongue rolling, licking/biting of pen fixtures)	Allogrooming Self-grooming Outcome not related to occurrence, frequency, and/or duration of NNOBs
Study Characteristics	Original empirical study Reports original data relating to direct measures of NNOBs in calves Peer-reviewed journal publication, including short communications English or German	Conference abstract or proceedings paper Language other than English or German Review papers Thesis papers Web article Surveys Questionnaires

### Data search, selection, management, and extraction

We initially used Web of Science to identify peer-reviewed relevant publications. A pilot search was conducted on April 4^th^, 2024 to test search terms. All searches included the following fixed search terms: TS = ((calf OR calves OR heifer) AND (dairy OR milking) AND (“non-nutritive oral behavio$r” OR “abnormal behavio$r” OR “abnormal oral behavio$r” OR “NNOB” OR “cross sucking” OR “cross-sucking” OR “oral stereo*” OR “non-nutritive sucking” OR “bite” OR “stere*”. To ensure literature saturation, we also added the following targeted intervention terms to the fixed-term search:

- AND (bucket OR nipple OR "AMF" OR Automat* milk fed*)
- *AND (ad lib OR milk allowance OR milk restricted)*
- AND (weaning OR weaned OR abrupt wean* OR gradual* wean*)
- *AND (frequency OR milk frequency OR feeding frequency)*
- *AND (environmental enrichment OR nutritional enrichment OR feeding enrichment OR physical enrichment OR “dry nipple” OR “dry teat” OR “artificial teat”)*
- *AND (hay OR “hay provision”)*
- AND (Group Size OR Group Age OR Group Comp*)

The initial search on Web of Science on April 4^th^, 2024, plus two additional searches using the fixed search terms on PubMed and the Agricultural & Environmental Science Database on October 29, 2024, resulted in 1320 studies. Additionally, reference lists of selected relevant review articles (S3 Table) addressing different aspects of dairy calf rearing were scanned for other papers that met our eligibility criteria listed articles. This way, we obtained another 17 studies for screening, resulting in 1337 studies in total. After removing duplicates (n = 489), 848 studies were screened in round one (abstract screening). During round one of screening, we excluded 718 studies (S4 Table), leaving 129 studies to move to round two (full text screening). In round two, we excluded an additional 45 studies (S5 Table) and retained 84 studies for data extraction (S6 Table). The PRISMA-P Flow chart of this process is included in S2 Table.

We had no restrictions on the date of publication. All search results were downloaded as a.RIS file and uploaded into Covidence [19] and Zotero [20] for storage.

All publications were managed in the Covidence database and subjected to two screening procedures. The first screening procedure reviewed the title and abstract of the publications; this information was used to make an initial decision whether the publication met our inclusion criteria (see Table 1). Author CRD screened 100% of the initial search results and author SK screened 50%; CRD and SK discussed any disagreements regarding inclusion and exclusion criteria. In cases of disagreement, DMW and MvK were asked to adjudicate. We recorded reasons for excluding studies when the study did not meet the inclusion criteria or there was uncertainty. The publications retained from round one of screening were compiled and subjected to round two of screening; completed solely by author CRD. The second round of screening consisted of reading the full text of each publication retained from round one and ensuring that publications met the inclusion criteria.

All search results were imported into Covidence. Duplicate articles were removed. Results from the screening and data extraction process were exported from Covidence and downloaded as an Excel form for data analysis. Zotero was used for backup storage of articles and reference management.

Pilot data collection was performed on a random selection of 10 articles by both CRD and SK (selection randomized by using the function “RAND” in Excel to assign randomized numbers to each publication followed by sorting the list from the smallest to largest random number and choosing the first 10 studies). Authors CRD and SK extracted pilot data to refine the final set of categories for optimal data collection techniques. Pilot data forms had the following information extracted:

1. **Study-level data**: Bibliography, title, abstract, language, year, country, study design.
2. **Population characteristics:** Number of farms in the study, type of farm (research, commercial, organic), breed of cattle, sex of animals.
3. **Farm management pre-treatment**: Duration of dam/calf contact, housing type, group size (if applicable), space allowance, milk feeding method, milk feeding frequency, milk volume, weaning method (if applicable), water access (y/n), hay provision (y/n), grain provision (y/n), roughage provision (y/n).
4. **Written descriptions of treatment and control methodology**.
5. **Farm management during treatment, if changed from pre-treatment:** Duration of dam/calf contact, housing type, group size (if applicable), space allowance, milk feeding method, milk feeding frequency, milk volume, weaning method (if applicable), water access (y/n), hay provision (y/n), grain provision (y/n), roughage provision (y/n).
6. **Intervention categories**: weaning methods, weaning duration, milk feeding frequency, milk feeding method, milk allowance, enrichment access, group size, group composition, hay provision, roughage provision, grain provision, water access, housing type (individual, pair, or group), feed supplementation, or human interaction.
7. **Outcome measures**: The outcome domain (which NNOBs were measured), the occurrence, frequency, and/or duration of NNOBs, metric used to characterize the results, the method of aggregation, and the timing of the outcome measurement.
8. **Behavioral observation methods**.
9. **Results written out in detail**.
10. **Conclusions from author:** the direction of effect of the results (positive, negative, neutral, or inconclusive/unclear).

On the basis of the pilot data, CRD and SK refined data extraction categories and then collected data using Covidence with the help of a customized data extraction template that included the finalized data extraction categories presented in Table 2. All authors agreed on the finalized data extraction categories. A copy of our data extraction template will be made publicly available at review publication. In cases of missing data not reported in the original article, the corresponding author(s) will be contacted to provide additional information or otherwise missing data coded as “not specified”. Primary outcomes recorded are the effect of the intervention applied on the occurrence, frequency, and/or duration of NNOBs.

**Table 2:** List of data extracted from the reviewed articles.

Information on article	Bibliography Title Authors Journal Year of publication Language Country
Information about the study design	Aims (are NNOBs a primary or secondary aim?) Study Design ◦ Type of design (i.e., randomized control trial, observational only, etc.) ◦ Quality of study (i.e., sampling method, experimental units specified (y/n) Number of Farms Farm Type (i.e., research, commercial, or organic) Sample size ◦ Sex ◦ Total number of calves ◦ Number of calves in treatment(s) and control(s) (number of experimental units) ◦ Total number of farms, number of calves per farm (where applicable) Breed Age of calves at enrollment and end of experimental period Housing type (i.e., individual, pair, or group) Group size of socially housed calves (if applicable) Space allowance during treatment(s) and control(s) Milk feeding method, allowance (volume), and frequency during the experimental period Calf to teat ratio (if applicable) Access to water, hay, or grain/feed/concentrate (y/n) Intervention categories applied (i.e., feeding method, enrichment, housing type, etc.) Ethical approval (y/n) NNOBs assessed Author definitions of NNOBs assessed Behavioral observation method used Video or in-person behavioral observation Time of behavioral observation in relation to milk meal
Information about general housing and feeding management	Duration spent with dam Housing type (i.e., individual, pair, or group) Group size/stocking density when applicable Enrichment access (y/n) Milk allowance, frequency, and feeding method Weaning method If calves had access to water, hay, or grain/feed/concentrate (y/n)
Outcome measures	Type of NNOBs assessed (e.g., cross-sucking, non-nutritive sucking on objects) Quantification of NNOBs (e.g., occurrence, duration, frequency) Non-NNOBs outcomes measures reported by authors (e.g., body weight, physiological measures)
Methods	Methodology written out in detail
Results	Results written out in detail Direction of effect of the results (positive, negative, neutral, or inconclusive/unclear) If the results were statistically significant (p < 0.05) Author’s interpretation of results
Conclusion	Conclusion written out in detail
Notes	Any notes from authors CRD or SK on the study

### Statistical analyses and quality assessment

After data extraction is conducted independently by authors CRD and SK on a randomized subset of publications (30%), inter-observer agreement levels will be analyzed using Cohen’s Kappa to ensure the reliability of data collection. Agreement on ≥  85% of collected data will be treated as an indication for no further refinements needed for the remaining data extraction. Inter-observer agreement below this threshold will require subsequent refining of the data extraction procedure. As a first means of synthesizing the data for comparison and analysis, we will create a table of study characteristics with the following information: Study ID, citation, study design, management intervention category, NNOB outcome domain (e.g., cross-sucking, bar sucking, etc.), outcome measure used, time frame of study, and if there is an effect or not (positive, negative, or no effect). This will allow us to descriptively assess study characteristics for further synthesis. We will then create subgroupings of studies based on the management intervention employed, and potentially other pertinent characteristics, such as study design.

As we have observed during preliminary data collection, some included studies do not provide effect measures and there is inconsistency overall in result reporting across studies. Therefore, we will first utilize the “description method” as a data analysis tool as outlined in the Cochrane handbook [21]. This method consists of vote counting based on the direction of effect with the goal to compare how many studies show beneficial effects versus detrimental or inconclusive effects. We have operationally defined the terms as follows: 1) positive effect: any effect which reduces the occurrence, frequency, and/or duration of any NNOBs, 2) negative effect: any effect which increases the occurrence, frequency, and/or duration, and 3) no effect: no change on the occurrence, frequency, and/or duration of NNOBs shown. We will then categorize effect estimates for all outcomes related to the occurrence, frequency, and/or duration of NNOBs according to these definitions. This will result in a count for each intervention category analysis (e.g., feeding method, enrichment provision, etc.) and outcome measure provided. Results of vote counting for each category of management intervention, alongside any available effect estimates, will be reported in a table. Additionally, we will visualize these results in a harvest plot with height representing the risk of bias for each intervention category (tall = low risk, medium =  some concerns, short =  high risk). We plan to conduct descriptive comparisons of study characteristics (e.g., how many studies examined hay provision, enrichment, etc.) and to provide these results in a table. To examine the difference in sample sizes, we plan to analyze the range of sample sizes included and record how many studies performed a power analysis. Finally, we will summarize our findings in sections of the manuscript based on management intervention and conclude with recommendations concerning interventions on dairy farms. A potential limitation of our planned statistical analysis is that this approach will not provide the same statistical robustness as when performing a meta-analysis. However, the body of literature we review is highly heterogeneous meaning that combining study findings might not always be appropriate [22]. Furthermore, NNOBs encompass a wide range of behaviours, which may be defined differently depending on the study. We will consult a statistician to discuss the possibility of performing a more robust meta-analysis analysis on our dataset (see S6 Table for number of studies within each theme). Where applicable, we will conduct either subgroup analyses or meta-regressions to examine how study characteristics influence the overall effects of interventions on NNOBs.

We will conduct a sensitivity analysis to confirm robustness of the findings by using the following methods: 1) repeat the analysis using only the studies rated as higher quality in the quality assessment, 2) repeat the analysis using only those studies that conducted inter-observer reliability testing on outcome measures. A risk of bias assessment of the systematic review itself will be completed with the ROBIS tool [23]. This tool will be used by two independent reviewers (DMW and MvK) covering four domains: 1) study eligibility criteria, 2)identification and selection of studies,3) data collection and study appraisal, and 4) synthesis and findings. We will summarize the confidence we have in each individual study included in the review by completing quality assessment forms using the ARRIVE (Animal Research: Reporting in Vivo Experiments) essential ten guidelines [24]. The ARRIVE guidelines promote transparency in the design and execution of animal studies to improve the reproducibility of research. These guidelines address: 1) Study design, 2) Sample size, 3) Inclusion and exclusion criteria, 4) Randomization, 5) Blinding, 6) Outcome measures, 7) Statistical methods, 8) Experimental animals, 9) Experimental procedures, and 10) Results. The signaling questions to be answered for each domain for each included study are provided in S7 Table. Each question was answered with “no”, “yes”, or “not applicable” and each domain has a text box for further explanation if needed. The ARRIVE signaling questions were intended to facilitate binary responses and each question was answered based on the information reported in the study. These questions are designed to provide an estimate of risk, scoring each study as: 1) low risk, 2) some concern, or 3) high risk. These results are provided in a risk of bias table using Robvis (https://mcguinlu.shinyapps.io/robvis/), showing each included study as a row and each domain as a column marked with one of three colored circles: 1) green (low risk), 2) yellow (some concern), or 4) red (high risk). Each study will also have a column for “overall risk of bias” based on the number of questions answered at a high or low risk of bias.

## Discussion

The methods described in this protocol will contribute to identifying studies that assess management factors relating to NNOBs in dairy calves. By following the PRISMA-P guidelines, we aim to minimize biases in data collection and provide a transparent review process. Various challenges and limitations may arise during the implementation of this review. For example, we expect heterogeneity between studies in management techniques, housing methods, and calf age and breed. Different studies will have likely measured different NNOBS, using different outcomes, and even measured the same outcomes in different ways. Therefore, we do not expect to conduct a meta-analysis. By systematically reviewing the effect of treatment, we hope to be able to make recommendations on management factors, the main motivation of our review. The robustness of our results will be verified through quality assessment and sensitivity analysis. As we will be drawing conclusions from peer-reviewed papers, our conclusion may be susceptible to a publication bias. We will record how many studies in our review have published null or negative results to estimate this bias and highlight knowledge gaps requiring further research.

We plan to disseminate results in a peer-reviewed, open-access journal publication. Any amendments to this protocol will be documented on The University of British Columbia’s digital repository (UBC Dataverse Collection) and uploaded where the protocol is registered (PLOS ONE). In the event of any amendment or termination, the rationale will be provided along with the date it is applied.

## Conclusion

This review protocol outlines the methodology for a systematic review evaluating the effects of farm management interventions on non-nutritive oral behaviors in dairy calves.

## Supporting information

S1 Table PRISMA-P 2015 checklist: recommended items to address in a systematic review protocol. (DOCX)

S2 Table PRISMA flow chart summarizing the results of our literature search (as February 5^th^ 2025). (PDF)

S3 Table Selected review articles for reference list scanning. (PDF)

S4 Table Excluded articles in round one of screening. (PDF)

S5 Table Excluded articles in round two of screening. (PDF)

S6 Table Included articles. (PDF)

S7 Table Quality assessment signaling questions from arrive 10 guidelines. (PDF)
