# Supplementary material for: Understanding non-nutritive oral behaviors in dairy calves (Bos taurus): A systematic review protocol
Source: PLoS One. 2025 Mar 20;20(3):e0319778. doi: 10.1371/journal.pone.0319778 (PMC11925274; doi:10.1371/journal.pone.0319778)

S2 Table. PRISMA Flow Chart Summarizing the Results of our Literature Search (as of February 5th, 2025).

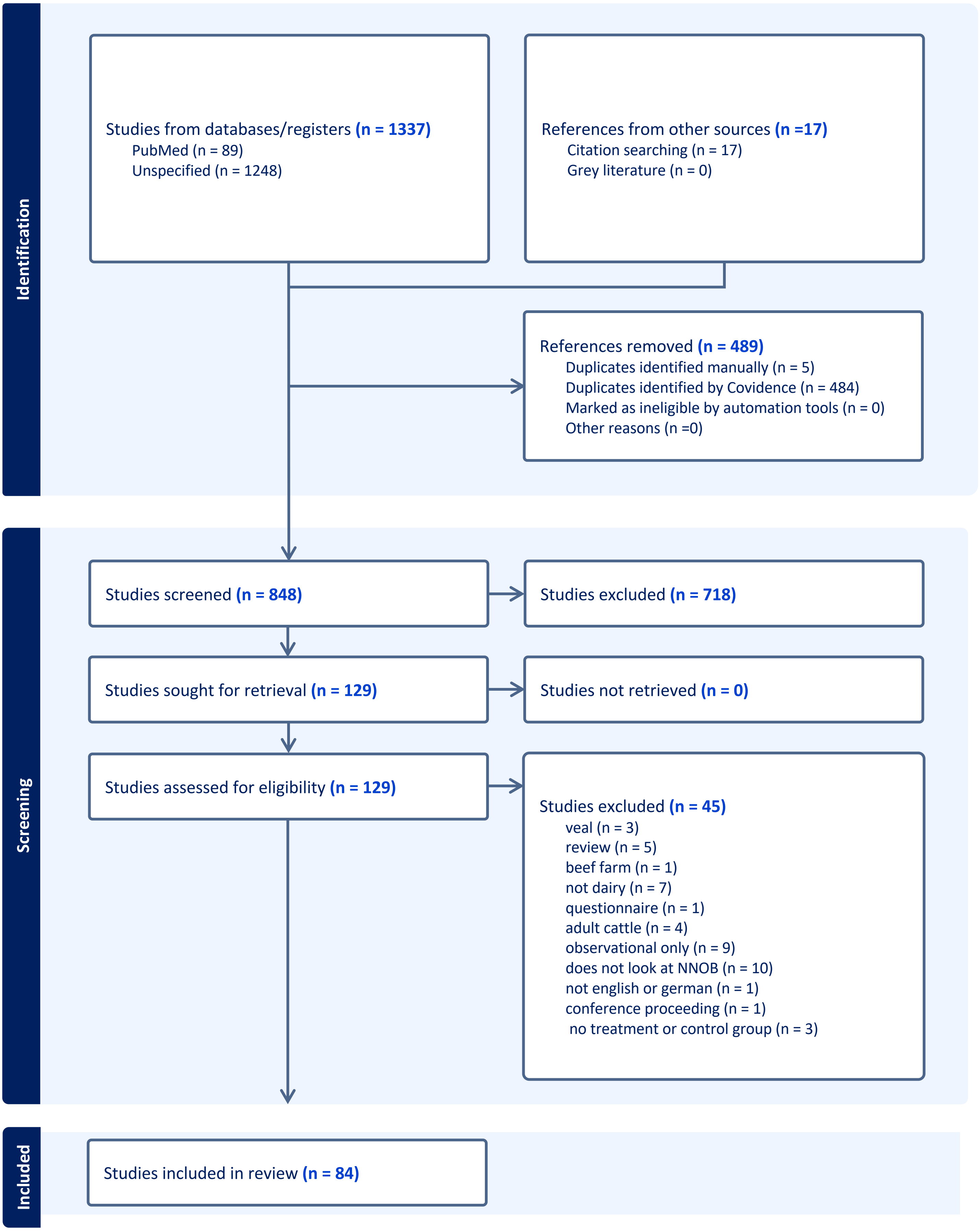

Supplement: S2 Table — (PDF) [file pone.0319778.s002.pdf]
