# Supplementary material for: Understanding non-nutritive oral behaviors in dairy calves (Bos taurus): A systematic review protocol
Source: PLoS One. 2025 Mar 20;20(3):e0319778. doi: 10.1371/journal.pone.0319778 (PMC11925274; doi:10.1371/journal.pone.0319778)
Supplement: S3 Table — (PDF) [file pone.0319778.s003.pdf]

S3 Table. Selected Review Articles for Reference List Scanning.

Author and year	Title	Citation
Kälber & Barth, 2014	Practical implications of suckling systems for dairy calves in organic production systems – a review	Kälber T, Barth K. Practical implications of suckling systems for dairy calves in organic production systems – a review. Landbauforschung. 2014;64(1):45-58
Lidfors & Isberg, 2003	Intersucking in dairy cattle—Review and questionnaire	Lidfors L, Isberg L. Intersucking in dairy cattle—Review and questionnaire. Appl Anim Behav Sci. 2003;80(3):207-231. doi:10.1016/S0168-1591(02)00215-0.
Meagher et al., 2019	Invited review: A systematic review of the effects of prolonged cow–calf contact on behavior, welfare, and productivity	Meagher RK, Beaver A, Weary DM, Von Keyserlingk MAG. Invited review: A systematic review of the effects of prolonged cow–calf contact on behavior, welfare, and productivity. J Dairy Sci. 2019;102(7):5765-5783. doi:10.3168/jds.2018-16021
Ridge et al., 2020	Effect of diet on non-nutritive oral behavior performance in cattle: A systematic review	Ridge E, Foster M, Daigle C. Effect of diet on non-nutritive oral behavior performance in cattle: A systematic review. Livest Sci. 2020;238:104063. doi:10.1016/j.livsci.2020.104063
Welk et al., 2023	Invited review: The effect of milk feeding practices on dairy calf behavior, health, and performance—A systematic review	Welk A, Otten ND, Jensen MB. Invited review: The effect of milk feeding practices on dairy calf behavior, health, and performance—A systematic review. J Dairy Sci. 2023. doi:10.3168/jds.2022-22900
Welk et al., 2024	Invited review: The effect of weaning practices on dairy calf performance, behavior, and health–a systematic review	Welk A, Neave HW, Jensen MB. Invited review: The effect of weaning practices on dairy calf performance, behavior, and health – a systematic review. J Dairy Sci. 2024. doi:10.3168/jds.2024-24521
