# Supplementary material for: Understanding non-nutritive oral behaviors in dairy calves (Bos taurus): A systematic review protocol
Source: PLoS One. 2025 Mar 20;20(3):e0319778. doi: 10.1371/journal.pone.0319778 (PMC11925274; doi:10.1371/journal.pone.0319778)
Supplement: S4 Table — (PDF) [file pone.0319778.s004.pdf]

S4 Table. Excluded Articles Round One of Screening.

Number	Title	First Author Year
1	Hay intake improves performance and rumen development of calves fed higher quantities of milk	Khan 2011
2	Invited review: Effects of group housing of dairy calves on behavior, cognition, performance, and health	Costa 2016
3	Maternal deprivation and the development of stereotypic behaviour	Latham 2008
4	Effect of restricted access time to pasture on dairy cow milk production, grazing behavior, and dry matter intake	Kennedy 2009
5	Management targets for maximising the short-term herbage intake rate of cattle grazing in Sorghum bicolor	Fonseca 2012
6	Changing concepts of farm animal welfare: bridging the gap between applied and basic research	Rushen 2003
7	Influence of weaning method on health status and rumen development in dairy calves	Roth 2009
8	Feeding behavior of calves fed small or large amounts of milk	Borderas 2009
9	Is rearing calves with the dam a feasible option for dairy farms? Current and future research	Johnsen 2016
10	Effects of sward height and concentrate supplementation on the ingestive behavior of spring-calving dairy-cows grazing grass clover swards	Rook 1994
11	Behavioural mechanisms of intake rate by heifers grazing swards of contrasting structures	Mezzalira 2014
12	Social Housing Improves Dairy Calves' Performance in Two Cognitive Tests	Gaillard 2014
13	The effects of feeding method, milk allowance and social factors on milk feeding behaviour and cross-sucking in group housed dairy calves	Jensen 2003
14	Short term fasting as a tool to design effective grazing strategies for lactating dairy cattle: a review	Chilibroste 2007
15	Invited review: A systematic review of the effects of prolonged cow-calf contact on behavior, welfare, and productivity	Meagher 2019
16	A 100 Year Review: Total mixed ration feeding of dairy cows	Schingoethe 2017
17	Bite dimensions and herbage intake by cattle grazing short hand-constructed swards	Ungar 1991
18	Nonnutritive oral activities and stress responses of veal calves in relation to feeding and housing conditions	Veissier 1998

19	Alpha-tocopherol concentration and stereoisomer composition in plasma and milk from dairy cows fed natural or synthetic vitamin E around calving	Meglia 2006
20	Eye white may indicate emotional state on a frustration-contentedness axis in dairy cows	Sandem 2002
21	A Survey of Management Practices That Influence Performance and Welfare of Dairy Calves Reared in Southern Brazil	Hötzel 2014
22	Effect of sward surface height and level of herbage depletion on bite features of cattle grazing Sorghum bicolor swards	Fonseca 2013
23	Review: Importance of colostrum supply and milk feeding intensity on gastrointestinal and systemic development in calves	Hammon 2020
24	Effect of feeding duration and rumen fill on behaviour in dairy cows	Lindström 2000
25	Dietary forage concentration and particle size affect sorting, feeding behaviour, intake and growth of Chinese holstein male calves	Muhammad 2016
26	Matching plant and animal processes to alter nutrient supply in strip-grazed cattle: Timing of herbage and fasting allocation	Gregorini 2008
27	Behavioural and physiological profiles following exposure to novel environment and social mixing in lambs	Miranda-delalama 2012
28	Stereotypies and cortisol secretion in heifers subjected to tethering	Redbo 1993
29	Timing of herbage allocation in strip grazing: Effects on grazing pattern and performance of beef heifers	Gregorini 2006
30	Effects of ruminal fill on short-term herbage intake rate and grazing dynamics of beef heifers	Gregorini 2007
31	Enantioselectivity in the disposition of ketoprofen and carprofen in man and animals	Delatour 1993
32	Resting and social behaviour of dairy heifers housed in slatted floor pens with different sized bedded lying areas	Nielsen 1997
33	Changes in ingestive behaviour of yearling dairy heifers due to changes in sward state during grazing down of rotationally stocked ryegrass or white clover pastures	Orr 2004
34	Relative bioavailability of all-rac and RRR vitamin E based on neutrophil function and total alpha-tocopherol and isomer concentrations in periparturient dairy cows and their calves	Weiss 2009
35	Intersucking in dairy cattle - review and questionnaire	Lidfors 2003
36	Effect of spring grazing date and stocking rate on sward characteristics and dairy cow production during midlactation	Kennedy 2007
37	The level of social contact affects social behaviour in pre-weaned dairy calves	Duve 2011

38	Epidemiology of flea infestation of ruminants in Libya	Kaal 2006
39	On the development of a monitoring scheme of buffalo welfare at farm level	Derosa 2005
40	Sward characteristics, grass dry matter intake and milk production performance is affected by timing of spring grazing and subsequent stocking rate	O'Donovan 2008
41	Gastrointestinal nematode infections and weight-gain in dairy replacement stock - 1st-year calves	Ploeger 1993
42	Bovine spongiform encephalopathy (bse) - the current situation and research	Bradley 1991
43	Post-grazing sward height imposed during the first 10 weeks of lactation: Influence on early and total lactation dairy cow production, and spring and annual sward characteristics	Ganche 2013
44	Ingestive behaviour of heifers grazing monocultures of ryegrass or white clover	Rutter 2002
45	Effect of the provision of a low-nutritive feedstuff on the behavior of dairy heifers limit fed a high-concentrate ration	Kitts 2011
46	Machine learning to detect behavioural anomalies in dairy cows under subacute ruminal acidosis	Wagner 2020
47	Once-a-day milking of multiparous Holstein cows throughout the entire lactation: milk yield and composition, and nutritional status	Rémond 2004
48	Defoliation pattern, foraging behaviour and diet selection by lactating dairy cows in response to sward height and herbage allowance of a ryegrass-dominated pasture	Tharmaraj 2003
49	Effect of Restricted Grazing Time on the Foraging Behavior and Movement of Tan Sheep Grazed on Desert Steppe	Chen 2013
50	Effect of herbage allowance on grazing behavior and productive performance of early lactation primiparous Holstein cows	Chilibroste 2012
51	Symposium review: Considerations for the future of dairy cattle housing: An animal welfare perspective	Beaver 2020
52	Detection of Bovine Coronavirus in Healthy and Diarrheic Dairy Calves	Gomez 2017
53	The role of solid feed amount and composition and of milk replacer supply in veal calf welfare	Webb 2015
54	Optimizing weaning strategies of dairy replacement calves	Bach 2010
55	Effects of mixing on drinking and competitive behavior of dairy calves	O'Driscoll 2006
56	Grazing behavior of rangeland beef-cattle differing in biological type	Funston 1991
57	D-Lactic Acidosis in Neonatal Ruminants	Lorenz 2014
58	Analysis of weaning-induced stress in Saanen goat kids	Magistrelli 2013

59	Foraging behaviour of beef heifers and ewes in natural grasslands with distinct proportions of tussocks	Bremm 2012
60	A barrier can reduce competition over teats in pair-housed milk-fed calves	Jensen 2008
61	Determination of pregastric lipase specificity in young ruminants	Villeneuve 1996
62	Current perspectives on the short- and long-term effects of conventional dairy calf raising systems: a comparison with the natural environment	Cantor 2019
63	Practical implications of suckling systems for dairy calves in organic production systems - a review	Kälber 2014
64	Restricting access time at pasture and time of grazing allocation for Holstein dairy cows: Ingestive behaviour, dry matter intake and milk production	Mattiauda 2013
65	The effect of breed and housing system on dairy cow feeding and lying behaviour	O'Driscoll 2009
66	Time budget, social and ingestive behaviours expressed by native beef cows in Mediterranean conditions	Braghieri 2011
67	Do Cytoplasmic Lipid Droplets Accumulate in Immature Oocytes from Over-Conditioned Repeat Breeder Dairy Heifers?	Awasthi 2010
68	Milking frequency and nutritional level affect grazing behaviour of dairy cows: A case study	O'Driscoll 2010
69	A brief report on effects of transfer from outdoor grazing to indoor tethering and back on urinary cortisol and behaviour in dairy cattle	Higashiyama 2007
70	Automatic weaning based on individual solid feed intake: Effects on behavior and performance of dairy calves	Benetton 2019
71	Ranking experts' preferences regarding measures and methods of assessment of welfare in dairy herds using Adaptive Conjoint Analysis	Lievaart 2011
72	Chiral inversion of (R)-ketoprofen: influence of age and differing physiological status in dairy cattle	Igarza 2002
73	Survey on housing, management and welfare of dairy cattle in tie-stalls in western Italian Alps	Mattiello 2005
74	Intake characteristics of perennial ryegrass varieties when grazed by yearling beef cattle under rotational grazing management	Orr 2005
75	Behaviour, welfare and productivity of dairy cattle	Rushen 1998
76	Early weaning: new insights on an ever-persistent problem in the dairy industry	Mikus 2020
77	Tongue-playing and heart rate in calves	Seo 1998
78	Effects of d-α-tocopherol and dietary energy on growth and health of preruminant dairy calves	Krueger 2014

79	Weight-gain and the course of some estimators of gastrointestinal nematode infection in calves during winter housing in relation to the level of exposure during the previous grazing season	Ploeger 1995
80	Evaluation of an innovative approach for sensory enrichment in zoos: semiochemical stimulation for captive lions (Panthera leo)	Martínez-Macipe 2015
81	Nitrogen and phosphorus retention and excretion in late-gestation dairy heifers	Hill 2007
82	Graphical analysis of sward depletion by grazing	Ungar 1992
83	Changes in the short-term intake rate of herbage by heifers grazing annual grasses throughout the growing season	Guzatti 2017
84	The effect of lying motivation on cow behaviour	Norring 2016
85	Intake and digestibility in heifers grazing a Dichanthium spp. Dominated pasture, at 14 and 28 days of regrowth	Boval 2007
86	Integration of natural behavior in housing systems	Lidfors 2005
87	Development of a micro-sward technique for determining intake characteristics of perennial ryegrass varieties	Orr 2005
88	Effect of diet on non-nutritive oral behavior performance in cattle: A systematic review	Ridge 2020
89	Identification of possible cow grazing behaviour indicators for restricted grass availability in a pasture-based spring calving dairy system	Werner 2019
90	Comparison of time budgets of growing Hereford bulls in an uninsulated barn and in extensive forest paddocks	Tuomisto 2008
91	Effect of group composition and feeding system on behaviour, production and health of dairy heifers in deep bedding systems	Hindhede 1999
92	Preliminary study on the effect of size of individual stall on the behavioural and immune reactions of dairy calves	Ferrante 1998
93	Effects of the provision of solid feeds enriched with protein or nonprotein nitrogen on veal calf growth, welfare, and slaughter performance	Brscic 2014
94	Short-term response in milk production, dry matter intake, and grazing behavior of dairy cows to changes in postgrazing sward height	Ganche 2014
95	Comparison of video and direct observation methods for measuring oral behaviour in veal calves	Tosi 2006
96	Sward structure and short-term herbage intake in Arachis pintoi cv. Belmonte subjected to varying intensities of grazing	Silva 2018
97	Growing dairy heifers prefer supplementary long straw when fed a nutrient-dense ration in a limited amount	Greter 2013
98	Intestinal histology of newborn goat kids fed lyophilized bovine colostrum	Nordi 2013

99	Comparison of protein and energy supplementation to mineral supplementation on feeding behavior of grazing cattle during the rainy to the dry season transition	Brandao 2016
100	Effect of different types of housing on behavior of Malpura lambs during winter in semi-arid tropical environment	De 2015
101	Insulin-like growth factor-I, passive immunity transfer, and stereological characteristics of small intestine of newborn calves	Pauletti 2007
102	Growth performance, behaviour, forestomach development and meat quality of veal calves provided with barley grain or ground wheat straw for welfare purpose	Cozzi 2002
103	The effect of grazing intensity on the performance of high-yielding dairy cows	Dale 2018
104	Ingestive Behavior of Heifers Supplemented with Glycerin in Substitution of Corn on Brachiaria brizantha Pasture	Facuri 2014
105	Relationship between Herbage Intake and Sward Structure of Grazed Temperate Grasses	Brink 2011
106	Grazing Behavior and Diet Preference of Beef Steers Grazing Adjacent Monocultures of Tall Fescue and Alfalfa: I. Spatial Allocation	Boland 2011
107	Differences in sward structure of ryegrass cultivars and impact on milk production of grazing dairy cows	Flores-Lesama 2006
108	Lameness in dairy cattle	Ward 2001
109	Natural and endotoxin-induced atresia of preantral and early antral follicles is characterized by DNA internucleosomal cleavage	Bosu 1996
110	Can access to an automated grooming brush and/or a mirror reduce stress of dairy cows kept in social isolation?	Mandel 2019
111	Oral behaviors of beef steers in pen and pasture environments	Ishiwata 2007
112	Influence of weaning on growth, health and behaviour of buffalo (Bubalus bubalis) calves	Singh 2019
113	Experimental Neospora caninum infection modifies trophoblast cell populations and plasma pregnancy-associated glycoprotein 1 and 2 dynamics in pregnant dairy heifers	Mur-Novales 2016
114	Effect of weaning on performance and behaviour of calves and their dams in dairy cows - A review	Kamboj 2013
115	Bio-discrimination of alpha-tocopherol stereoisomers in rearing and veal calves fed milk replacer supplemented with all-rac-alpha-tocopheryl acetate	Dersjant-Li 2009
116	Effect of type of supplement offered out of parlour on grazing behaviour and performance by lactating dairy cows grazing continuously stocked grass swards	Gibb 2002

117	Research on the Schizont cell culture vaccine against Theileria annulata infection in Xinjiang, China	Guo 1997
118	Views of Western Canadian dairy producers on calf rearing: An interview-based study	Russell 2022
119	Overview of common practices in calf raising facilities	Machado 2022
120	Pharmacokinetic Parameters of (R)-(−) and (S)-(+)-Flurbiprofen in Dairy Bovines	Igarza 2006
121	Skin stimulated induction of mouth movements in cattle	Simonsen 1994
122	Effects of the anti-sucking device «suckstop Müller» on calf behavior	Bisang 2022
123	Wound lesions caused by ear tagging in unweaned calves: assessing the prevalence of wound lesions and identifying risk factors	Hayer 2022
124	Effect of Different Rearing during the Milk-Feeding Period on Growth of Dairy Calves	Broucek 2020
125	Ingestive behavior of pastured crossbred dairy cows offered different supplement types	Mendes 2012
126	Granulo-pustular vulvovaginitis ("Jackal bite") an emerging disease: Mycoplasma bovigenitalium and M. Canadense infection of dairy cattle in Israel	Brenner 2009
127	Function of Tongue-Playing of Cattle in Association With Other Behavioral and Physiological Characteristics	Ishiwata 2008
128	Effect of the level of concentrate supplementation on the voluntary intake and feeding behavior of dairy cows on spring grazing	Riquelme 2008
129	A video and acoustic methodology to map bite placement at the patch scale	Griffiths 2006
130	Effect of a milk byproduct-based calf starter feed on dairy calf nutrient consumption, rumen development, and performance when fed different milk levels	Parsons 2022
131	Feeding concentrate pellets enriched by natural vitamin E keeps the plasma vitamin E above the critical level in calves post-weaning	Lashkari 2021
132	Grazing behaviour of dairy cattle in relation to genetic selection for milk production	Fuerst-Waltl 1997
133	Effects of Heat-Stress on Oocyte Number and Quality and In Vitro Embryo Production in Holstein Heifers	Çizmeci 2022
134	Response to different sources of vitamin E orally injected and to various doses of vitamin E in calf starter on the plasma vitamin E level in calves around weaning	Lashkari 2022
135	Milk performance and grazing behaviour of dairy cows in response to pasture allowance	Zanine 2019
136	Characteristics of a group of 21 patients allergic to meat by sensitization to alpha-Gal allergens	Drouet 2016

137	Experimental infection of Holstein cows and calves with EHDV-7 and preliminary evaluation of different inoculation methods	Ruder 2015
138	Empirical assessment of short-term preferences of tropical forages by crossbred bull calves	Komwihangilo 2007
139	Determining the pre-grazing sward height of Kikuyu grass (Cenchrus clandestinus - Hochst. Ex Chiov.) For optimizing nutrient intake rate of dairy heifers	Gómez 2022
140	Relationship between Temperate Grass Sward Characteristics and the Grazing Behavior of Dairy Heifers	Soder 2022
141	Performance and feeding behavior of Holstein and Holstein x Gyr crossbred heifers grazing temperate forages	Abreu 2022
142	Twenty-five-centimeter pre-grazing canopy height in palisade grass and forage peanut	Ferreira 2022
143	A methodological approach to compare continuous and instantaneous sampling and two methods to deal with animals out of sight on dairy cattle behavior and interaction with their calf in the first hours post-partum	Manfrè 2024
144	Is there a right time for dairy Alpine goat kid weaning: How does the weaning age of dairy Alpine goat kids affect their growth and behavior?	Bélanger-Naud 2024
145	Evaluation of behavior in veal calves fed milk containing different levels of hempseed cake (Cannabis sativa L.)	Arango 2023
146	Dairy farmer and farm staff attitudes and perceptions regarding daily milk allowance to calves	Svensson 2023
147	Invited review: The effect of milk feeding practices on dairy calf behavior, health, and performance-A systematic review	Welk 2023
148	Pre-Implantation Bovine Embryo Evaluation-From Optics to Omics and Beyond	Rabel 2023
149	Welfare implications on management strategies for rearing dairy calves: A systematic review. Part 2-Social management	Carulla 2023
150	Changes in amount and length of periods of stereotypic behavior in Jersey cows with and without access to pasture	Demba 2023
151	Effect of mother bonded rearing on growth, health and physiological state of Murrah buffalo calves	Jamwal 2022
152	Incisionless Single Intralingual Suture for Management of Milk Vice in Cattle	Fazili 2022
153	Clinical examination of cattle. Part 2: calves, technology and ancillary testing	Sherwin 2022
154	Effect of a High Welfare Floor and a Concrete Slatted Floor on the Growth Performance, Behavior and Cleanliness of Charolais and Limousin Heifers: A Case Study	Leskovec 2022

155	Optimal rates for feeding front for cows during the milking and midlactation period when contained at dairy complexes of the industrial type	Shamonina 2022
156	Ingestive behavior of Girolando heifers in integrated crop, livestock (ICL), and forestry (ICLF) systems	Souza 2021
157	Management Tips for Calves Fed With Automated Milk Feeders	Nielsen 2012
158	Comparison of two conventional restricted daily milk allowance methods in dairy calf rearing with respect to growth and behavioural responses - II. Behavioural responses	Tapki 2007
159	Computer-controlled milk feeding of calves; the effect of precise milk allocation	Halachmi 2005
160	Alpha-tocopherol concentration and stereoisomer composition in plasma and milk from dairy cows fed natural or synthetic vitamin E around calving	Meglia 2005
161	Relative bioavailability of all-rac and RRR vitamin E based on neutrophil function and total alpha-tocopherol and isomer concentrations in periparturient dairy cows and their calves	Weiss 2009
162	Evaluation of an innovative approach for sensory enrichment in zoos: semiochemical stimulation for captive lions (Panthera leo)	Martínez-Macipe 2015
163	Invited review: The effect of milk feeding practices on dairy calf behavior, health, and performance-A systematic review	Welk 2023
164	Effect of space allowance in deep bedding systems on resting behaviour, production, and health of dairy heifers	Mogensen 1997
165	Intake and digestibility in heifers grazing a Dichanthium spp. Dominated pasture, at 14 and 28 days of regrowth	Boval 2007
166	Invited review: Effects of group housing of dairy calves on behavior, cognition, performance, and health	Costa 2016
167	Influence of weaning on growth, health and behaviour of buffalo (Bubalus bubalis) calves	Singh 2019
168	A 100-Year Review: Total mixed ration feeding of dairy cows	Schingoethe 2017
169	Chiral inversion of (R)-Ketoprofen: Influence of age and differing physiological status in dairy cattle	Igarza 2002
170	Pharmacokinetic parameters of (R)-(-) and (S)-(+)-flurbiprofen in dairy bovines	Igarza 2006
171	Effects of Heat-Stress on Oocyte Number and Quality and In Vitro Embryo Production in Holstein Heifers	Cizmeci 2022
172	Oregano Extract Added into the Diet of Dairy Heifers Changes Feeding Behavior and Concentrate Intake	Kolling 2016

173	Effects of hay grass level and its physical form (full length vs. Chopped) on standing time, drinking time, and social behavior of calves	MAR Muhammad Aziz-ur-Rahman 2017
174	Effect of concentrate feeder design on performance, eating and animal behavior, welfare, ruminal health, and carcass quality in Holstein bulls fed high-concentrate diets	Verdú, 2015
175	Fattening Holstein heifers by feeding high-moisture corn (whole or ground) ad libitum separately from concentrate and straw1	Devant 2015
176	Effects of dietary nonstructural carbohydrates and protein sources on feeding behavior of tethered heifers fed high-concentrate diets	Rotger 2006
177	Short communication: Pair housing dairy calves in modified calf hutches.	Whalin 2018
178	Impacts of self- and cross-sucking on cattle health and performance.	Mahmoud 2016
179	Symposium review: Considerations for the future of dairy cattle housing: An animal welfare perspective.	Beaver 2020
180	Influences of human contact following milk-feeding on nonnutritive oral behavior and rest of individual and pair-housed dairy calves during weaning.	Doyle 2023
181	Evaluation of behavior in veal calves fed milk containing different levels of hempseed cake (Cannabis sativa L.).	Arango 2023
182	Invited review: A systematic review of the effects of prolonged cow-calf contact on behavior, welfare, and productivity.	Meagher 2019
183	Experimental Neospora caninum infection modifies trophoblast cell populations and plasma pregnancy-associated glycoprotein 1 and 2 dynamics in pregnant dairy heifers.	Mur-Novales 2016
184	Characterization of a model to induce hyperlipidemia in feed-restricted dairy cows.	Arshad 2024
185	Determining the pre-grazing sward height of Kikuyu grass (Cenchrus clandestinus - Hochst. Ex Chiov.) For optimizing nutrient intake rate of dairy heifers.	Marín Gómez 2022
186	Some pharmacokinetic parameters of R-(-)- and S-(+)-ketoprofen: the influence of age and differing physiological status in dairy cattle.	Igarza 2004
187	Effects of amount of milk, milk flow and access to a rubber teat on cross-sucking and non-nutritive sucking in dairy calves.	Jung 2001
188	Relative bioavailability of all-rac and RRR vitamin E based on neutrophil function and total alpha-tocopherol and isomer concentrations in periparturient dairy cows and their calves.	Weiss 2009
189	Indigestion in young calves. IV. Lesions of ruminal papillae in young calves fed barley and barley plus hay.	Landsverk 1978

190	Behavior, Intake, Digestion and Milk Yield of Early Lactation Holstein Dairy Cows with Two Levels of Environmental Exposure and Feeding Strategy.	Mendez 2024
191	Gastrointestinal nematode infections and weight gain in dairy replacement stock: first-year calves.	Ploeger 1993
192	Alpha-tocopherol concentration and stereoisomer composition in plasma and milk from dairy cows fed natural or synthetic vitamin E around calving.	Meglia 2006
193	Effects of the presence of grazing-experienced heifers on the development of foraging behavior at the feeding station scale for first-grazing season calves.	Shingu 2017
194	Histological measurement of fat content of liver of dairy cows.	Collins 1985
195	Bio-discrimination of alpha-tocopherol stereoisomers in rearing and veal calves fed milk replacer supplemented with all-rac-alpha-tocopheryl acetate.	Dersjant-Li 2009
196	Use of nonergot alkaloid-producing endophytes for alleviating tall fescue toxicosis in stocker cattle.	Parish 2003
197	Arginine infusion stimulates prolactin, growth hormone, insulin, and subsequent lactation in pregnant dairy cows.	Chew 1984
198	Effects of administration of mycobacterium cell wall fraction during the periovulatory period on embryo development following superovulation in virgin dairy heifers.	Brown 2024
199	Effect of recovery period of mixture pasture on cattle behaviour, pasture biomass production and pasture nutritional value.	Pereira 2020
200	Dietary vitamin A modulates the concentrations of RRR-alpha-tocopherol in plasma lipoproteins from calves fed milk replacer.	Ametaj 2000
201	A correlated biochemical and stereological study of periparturient fatty liver in the dairy cow.	Collins 1980
202	[The protein-synthesizing function of the liver in dairy cows fed feed with the addition of various urea preparations and different doses of urea].	Kwiatkowski 1986
203	[Comparative enantioselectivity of the disposition of two non-steroidal anti-inflammatory agents, ketoprofen and carprofen, in man and animals].	Delatour 1993
204	Cerebral ventricle cannulation in the calf.	Hedlund 1977
205	Changes of tocopherols in blood serum of cows fed hay or silage.	Lynch 1983
206	Genetic legacy and adaptive signatures: investigating the history, diversity, and selection signatures in Rendena cattle resilient to eighteenth century rinderpest epidemics	Somenzi 2024
207	Finding biomarkers of experience in animals	Babington 2024

208	Epidemiological Study of Lumpy Skin Disease Outbreaks in Egypt Based on Viral Isolation and Molecular Detection	Elsheikh 2024
209	Camera collars reveal macronutrient balancing in free -ranging male moose during summer	Spitzer 2024
210	Taphonomic signatures of early scavenging by black and turkey vultures	Wahl 2024
211	Investigation of Bovine Disease and Events through Machine Learning Models	Nadeem 2024
212	Transition milk or milk replacer powder as waste milk supplements to cold-stressed neonatal Holstein dairy calves: Effects on performance, feeding behavior, and health	Moradi 2024
213	The effect of rest duration on the physical and chemical quality of Brahman-Cross beef	Gaznur 2024
214	Comparative production performance and rumen bacterial diversity of fattening beef cattle supplemented with different levels concentrated feed	Han 2024
215	Effect of Cryopreservation on Conception Rates of in-vitro Produced Sahiwal Embryos	Jakkali 2024
216	Measurement of the Direct Impact of Hematophagous Flies on Feeder Cattle: An Unexpectedly High Potential Economic Impact	Boonsaen 2024
217	Canopy Characteristics of Gamba Grass Cultivars and Their Effects on the Weight Gain of Beef Cattle under Grazing	Braga 2024
218	The Impact of Storytelling about an Innovative and Sustainable Organic Beef Production System on Product Acceptance, Preference, and Satisfaction	Najdek 2024
219	The Short-Term Effects of Altering Milking Intervals on Milk Production and Behavior of Holsteins Milked in an Automated Milking System	Davis 2024
220	A Clean and Health-Care-Focused Way to Reduce Indoor Airborne Bacteria in Calf House with Long-Wave Ultraviolet	Ding 2024
221	Advancements in Real-Time Monitoring of Enteric Methane Emissions from Ruminants	O’Connor 2024
222	Good Handling Practices Have Positive Impacts on Dairy Calf Welfare	Silva-Antunes 2024
223	Combining Double-Dose and High-Dose Pulsed Dapsone Combination Therapy for Chronic Lyme Disease/Post-Treatment Lyme Disease Syndrome and Co-Infections, Including Bartonella: A Report of 3 Cases and a Literature Review	Horowitz 2024
224	An Integrated Pest Management Strategy Approach for the Management of the Stable Fly Stomoxys calcitrans (Diptera: Muscidae)	Gonzalez 2024

225	The Impact of Early-Life Cecal Microbiota Transplantation on Social Stress and Injurious Behaviors in Egg-Laying Chickens	Fu 2024
226	Combinations of Lemongrass and Star Anise Essential Oils and Their Main Constituent: Synergistic Housefly Repellency and Safety against Non-Target Organisms	Soonwera 2024
227	Preserving Ethnoveterinary Medicine (EVM) along the Transhumance Routes in Southwestern Angola: Synergies between International Cooperation and Academic Research	Solazzo 2024
228	Pasture Access Effects on the Welfare of Dairy Cows Housed in Free-Stall Barns	Petrean 2024
229	Computer Vision-Based Measurement Techniques for Livestock Body Dimension and Weight: A Review	Ma 2024
230	Development of a roadmap for action for the project More Welfare: towards new risk assessment methodologies and harmonised animal welfare data in the EU	Paulović 2024
231	Vaccines for Bovine Mastitis are Safe and Efficacious in Laboratory Animals	Bilal Ahmed Shah 2023
232	Abstracts from the 132nd Meeting of the Tennessee Academy of Science November 18, 2022	Anonymous 2023
233	Evaluation of Holstein cows with different tongue-rolling frequencies: stress immunity, rumen environment and general behavioural activity	Sun 2023
234	The age at first consumption of forage in calves and its effect on growth and rumination in the short- and long-term	Xiao 2023
235	Seroepidemiological investigation of Crimean Congo hemorrhagic fever virus in livestock in Uganda, 2017	Nyakarahuka 2023
236	Epizootic Hemorrhagic Disease (EHD) – Systematic Literature Review report	Mariana Avelinode Souza Santos 2023
237	Exploring Ethno-veterinary Practices for Livestock Diseases: A Survey-Based Approach	Krishna 2023
238	Silvopastoral system: persistence of Andropogon grass and grazing behavior of goats	Zambrano 2023
239	Milk-Clotting and Proteolytic Properties of a Partially Purified Pepsin from Yellowfin Tuna (Thunnus albacares) and its Potential for Cheesemaking	Osuna-Ruiz 2023
240	Comparative Evaluation of Developmental Competence of Immature Cattle Oocytes in Three Different Culture Media	Karmali 2023
241	Dietary intake of α-ketoglutarate ameliorates α-synuclein pathology in mouse models of Parkinson's disease	Zhang 2023
242	Response to novel feed in dairy calves is affected by prior hay provision and presentation method	Morrow 2023

243	Effect of pre-grazing herbage mass and post-grazing sward height on herbage production and intake and performance of suckler-bred steers within a weanling-to-beef production system	Doyle 2023
244	Tropical grass silages with spineless cactus in diets of Holstein × Zebu heifers in the semiarid region of Brazil	Cordeiro 2023
245	Welfare of calves	Nielsen 2023
246	Edible insects: Tendency or necessity (a review)	Papastavropoulou 2023
247	Surveillance plan proposal for early detection of zoonotic pathogens in ruminants	Schüpbach 2023
248	Extensive literature search on mineral oil hydrocarbons	Licht 2023
249	'I owe it to the animals': The bidirectionality of Swiss alpine farmers' relational values	Chapman 2023
250	A Korean Cattle Weight Prediction Approach Using 3D Segmentation-Based Feature Extraction and Regression Machine Learning from Incomplete 3D Shapes Acquired from Real Farm Environments	Chang Gwon Dang 2023
251	Biting Midges (Diptera: Ceratopogonidae) as Vectors of Viruses	Kampen 2023
252	Applications of Enzyme Technology to Enhance Transition to Plant Proteins: A Review	Gouseti 2023
253	Epidemiology of Zoonotic Coxiella burnetii in The Republic of Guinea	Ohlopkova 2023
254	Physicochemical, Microbiological and Sensory Characteristics of White Brined Cheese Ripened and Preserved in Large-Capacity Stainless Steel Tanks	Massouras 2023
255	Unlocking Lethal Dingo Management in Australia	Boronyak 2023
256	Grassland Ecology and Ecosystem Management for Sustainable Livestock Performance	Harmon 2023
257	Challenges of Pasture Feeding Systems—Opportunities and Constraints	Wróbel 2023
258	Tick Diversity and Distribution of Hard (Ixodidae) Cattle Ticks in South Africa	Makwarela 2023
259	Development of Thresholds to Predict Grazing Behaviour of Dairy Cows from Motion Sensor Data and Application in a Pasture-Based Automatic Milking System	Cullen 2023
260	A Non-Contact Cow Estrus Monitoring Method Based on the Thermal Infrared Images of Cows	Wang 2023
261	Precision Livestock Farming Applications (PLF) for Grazing Animals	Tzanidakis 2023
262	Recommendations and technical specifications for sustainable surveillance of zoonotic pathogens where wildlife is implicated	Gavier-Widen 2023
263	Cultivation and Uses of Moringa oleifera as Non-Conventional Feed Stuff in Livestock Production: A Review	Abdoun 2023

264	Evaluating accurate and efficient sampling strategies designed to measure social behavior and brush use in drylot housed cattle	Lozada 2023
265	Implementation of food matrix effects into chemical food contaminant risk assessment	Ana-andreeacioca 2022
266	The Dasgupta Review and the Problem of Anthropocentrism	Treich 2022
267	Effect of weaning and feed provision times on the performance and several behavioural traits of post-weaning lambs	Goliomytis 2022
268	Observation on dromedary (Camelus dromedarius) welfare and husbandry practices among nomadic pastoralists	Diolimaurizio 2022
269	A blood digestion scoring method for poultry red mites, Dermanyssus gallinae	Ma 2022
270	Effect of dietary inclusion of licuri cake on intake, feeding behavior, and performance of feedlot cull cows	Silva 2022
271	Welfare of cattle during transport	Nielsen 2022
272	Welfare of pigs on farm	Nielsen 2022
273	The application of allostasis and allostatic load in animal species: A scoping review	Seeley 2022
274	Effect of forage to concentrate ratio on growth performance and feeding behavior of Thalli lambs	Chishti 2022
275	Feeding behavior of post-weaned crossbred steers supplemented in the dry season of the year	Lins 2022
276	Nasopulmonary mites (Acari: Halarachnidae) as potential vectors of bacterial pathogens, including Streptococcus phocae, in marine mammals	Pesapane 2022
277	A review on water intake in dairy cattle: associated factors, management practices, and corresponding effects	Singh 2022
278	Characterization of grazing behaviour microstructure using point-of-view cameras	Sales-Baptista 2022
279	Sensor based time budgets in commercial Dutch dairy herds vary over lactation cycles and within 24 hours	Hut 2022
280	Judgement bias of group housed gestating sows predicted by behavioral traits, but not physical measures of welfare	Horback 2022
281	Occurrence of ticks and tick-borne mixed parasitic microbiota in cross-bred cattle in District Lahore, Pakistan	Hasan 2022
282	Immunomodulatory Effects of Macrolides Considering Evidence from Human and Veterinary Medicine	Blondeau 2022
283	Embryo Morphokinetic Activity Evident in Short Videos of In Vitro Bovine Embryos	Wells 2022
284	Mastitis: Impact of Dry Period, Pathogens, and Immune Responses on Etiopathogenesis of	Egyedy 2022

	Disease and its Association with Periparturient Diseases	
285	The Middle Eastern Cousin: Comparative Venomics of Daboia palaestinae and Daboia russelii	Rrsenjilaxme 2022
286	Live Weight Prediction of Cattle Based on Deep Regression of RGB-D Images	Ruchay 2022
287	Techniques Used to Determine Botanical Composition, Intake, and Digestibility of Forages by Ruminants	Pepeta 2022
288	A Model for Iberian Wolf (Canis lupus signatus, Cabrera 1907) Predation Risk Assessment on Cattle in the Central System (Spain)	Velázquez 2022
289	Does the Rearing Management following by Charolais Cull Cows Influence the Qualities of Carcass and Beef Meat?	Soulat 2022
290	Nitrogen Use Efficiency and Partitioning of Dairy Heifers Grazing Perennial Ryegrass (Lolium perenne L.) Or Pasture Brome (Bromus valdivianus Phil.) Swards during Spring	Beltran 2022
291	Evaluating the Shelf Life and Sensory Properties of Beef Steaks from Cattle Raised on Different Grass Feeding Systems in the Western United States	Duarte 2022
292	Predicting the Feed Intake of Cattle Based on Jaw Movement Using a Triaxial Accelerometer	Ding 2022
293	Protective Effect of Alkaline Phosphatase Supplementation on Infant Health	Wu 2022
294	Integrative Alternative Tactics for Ixodid Control	Showler 2022
295	Contribution of Precision Livestock Farming Systems to the Improvement of Welfare Status and Productivity of Dairy Animals	Simitzis 2022
296	Cow metabolic status assessed from fat/protein ratio in milk affected ovarian response and number of transferable embryos after superovulation	Stádník 2022
297	Decision Support System (DSS) for Managing a Beef Herd and Its Grazing Habitat's Sustainability: Biological/Agricultural Basis of the Technology and Its Validation: Biological/Agricultural Basis of the Technology and Its Validation	Asher 2022
298	Grazing Management Targets for Tangolagrass Pastures	Mocelin 2022
299	Effects of different planes of milk feeding and milk total solids concentration on growth, ruminal fermentation, health, and behavior of late weaned dairy calves during summer	Shiasi Sardoabi 2021
300	Determination of some body measurements of camels with three-dimensional modeling method (3D)	Çağlı 2021
301	New animal-based measures to assess welfare in dromedary camels	Menchetti 2021

302	Risk factors associated with Ctenocephalides felis flea infestation of peri-urban goats: a neglected parasite in an under-appreciated host	Dahm 2021
303	Pesisir cattle superovulation with various dosage of Follicle Stimulating Hormone (FSH) on embrio production	Afriani 2021
304	Prediction of dry matter intake by meat sheep on tropical pastures	Chaves 2021
305	Changes in tail posture detected by a 3D machine vision system are associated with injury from damaging behaviours and ill health on commercial pig farms	D'Eath 2021
306	Does Australian oaten hay improve Chinese dairy cow performance?	Liang 2021
307	Effect of adjunct starter culture on the quality of reduced fat, white, brined goat cheese: part I. Assessment of chemical composition, proteolysis, lipolysis, texture and sensory attributes	Zaravela 2021
308	Why and how farmers manage mixed cattle‚Äìsheep farming systems and cope with economic, climatic and workforce-related hazards	Mugnier 2021
309	Bottlenose dolphin habitat and management factors related to activity and distance traveled in zoos and aquariums	Lauderdale 2021
310	Safety assessment of titanium dioxide (E171) as a food additive	Younes 2021
311	Dromedary camel health care practices reported by caretakers working at a permanent market	Padalino 2021
312	Arctic Change 2020 Conference Abstracts	
313	Growth and development of replacement heifers depending on the origin	Gorelik 2021
314	Health and welfare in organic livestock production systems - a systematic mapping of current knowledge	Presto 2021
315	Impact of Haematobia exigua (Buffalo Fly) in Cattle in Namakkal Region, Tamil Nadu	Anbarasi 2021
316	Epidemiological Studies on Physical, Chemical, Zoonotic and Psychological Hazards among Veterinarians	Parmar 2021
317	Recycling biological waste using the fly Hermetia illucens, environmental risks and biosafety for Russia	Syromyatnikov 2021
318	ASAS-NANP SYMPOSIUM: Applications of machine learning for livestock body weight prediction from digital images	Wang 2021
319	Advancements in sensor technology and decision support intelligent tools to assist smart livestock farming	Tedeschi 2021
320	Socio-economic constraints on camel production in Pakistan’ss extensive pastoral farming	Asim 2021

321	A knowledge, attitudes, and practices study on ticks and tick-borne diseases in cattle among farmers in a selected area of eastern Bhutan	Namgyal 2021
322	Herbage accumulation, canopy structure and tiller morphology of marandu palisadegrass growing in open pasture and in silvopasture	Nascimentohl 2021
323	Productive and reproductive performance and blood chemistry on grazing Brahman replacement heifers supplemented with fatty acids and protein	Bellofaria 2021
324	Efficient induction and sustenance of pluripotent stem cells from bovine somatic cells	Viju Vijayan Pillai 2021
325	Nutritional Modulation of the Immune Response Mediated by Nucleotides in Canine Leishmaniosis	Segarra 2021
326	Nanotechnology as a Processing and Packaging Tool to Improve Meat Quality and Safety	Lamri 2021
327	Effect of Raising Dairy Heifers on Their Performance and Reproduction after 12 Months	Uhrincat 2021
328	Effect of the Initial Time of Providing Oat Hay on Performance, Health, Behavior and Rumen Fermentation in Holstein Female Calves	Li 2021
329	Detecting Dairy Cow Behavior Using Vision Technology	Mcdonagh 2021
330	Tick-Borne Encephalitis Vaccination Protects from Alimentary TBE Infection: Results from an Alimentary Outbreak	Chitimia-Dobler 2021
331	The Deviation between Dairy Cow Metabolizable Energy Requirements and Pasture Supply on a Dairy Farm Using Proximal Hyperspectral Sensing	Duranovich 2021
332	Ticks and Tick-Borne Diseases of Livestock in the Middle East and North Africa: A Review	Perveen 2021
333	Quark-Type Cheese: Effect of Fat Content, Homogenization, and Heat Treatment of Cheese Milk	Lepesioti 2021
334	Farming systems in sheep rearing: Impact on growth and reproductive performance, nutrient digestibility, disease incidence and heat stress indices	Karthik 2021
335	Behavior assessment and applications for BRD diagnosis: preweaned dairy calves	Cramer 2020
336	Application of platelet-rich plasma in the in vitro production of bovine embryos	Ramos-Deus 2020
337	Performance of Purunã beef heifers and pasture productivity in a long-term integrated crop-livestock system: the effect of trees and nitrogen fertilization	Pontes 2020
338	Calf production of Bali cows in cattle-oil palm plantation integration system in Riau Province Indonesia	Baliarti 2020

339	Molecular detection of pathogens in ticks associated with domestic animals from the Colombian Caribbean region	Cotes-Perdomo 2020
340	Effects of hay provision and presentation on cognitive development in dairy calves	Horvath 2020
341	Growth Potential of Camelus dromedarius Calves Reared under Intensive and Extensive Feeding Management Systems	Asim Faraz 2020
342	Is similarity in Major Histocompatibility Complex (MHC) associated with the incidence of retained fetal membranes in draft mares? A cross-sectional study	Jaworska 2020
343	Methodology for experimental and observational animal studies in cow-calf contact systems	Deoliveira 2020
344	Systems for evaluation of welfare on dairy farms	Krueger 2020
345	High laboratory mouse pre-weaning mortality associated with litter overlap, advanced dam age, small and large litters	Morello 2020
346	Effect of supplementation with tree foliage on in vitro digestibility and fermentation, synthesis of microbial biomass and methane production of cattle diets	Albores-Moreno 2020
347	Studded leather collars are very effective in protecting cattle from leopard (Panthera pardus) attacks	Khorozyan 2020
348	Importance of livestock diseases identified using participatory epidemiology in the highlands of Ethiopia	Gizawsolomon 2020
349	Beef cattle responses to pre-grazing sward height and low level of energy supplementation on tropical pastures	Dórea 2020
350	Molecular detection of Coxiella burnetii in livestock farmers and cattle from Magdalena Medio in Antioquia, Colombia	Ruth Cabrera Orrego 2020
351	Insect-repelling behavior in goitered gazelles: responses to biting fly attack	Blank 2020
352	Hashtag hijacking and crowdsourcing transparency: social media affordances and the governance of farm animal protection	Rodak 2020
353	Short Communication - Comparison of Growth Rate of Camelus dromedarius Calves Reared under Open Grazing/Browsing and Stall Fed System	Asimfaraz 2020
354	Proceedings of the British Society of Animal Science	
355	A genetic and immunological comparison of tick-resistance in beef cattle following artificial infestation with Rhipicephalus ticks	Marima 2020
356	Cattle adapted to tropical and subtropical environments: social, nutritional, and carcass quality considerations	Cooke 2020
357	Evaluation of Biological, Textural, and Physicochemical Parameters of Panela Cheese Added with Probiotics	Parra-Ocampo 2020

358	Tick Fauna and Associated Rickettsia, Theileria, and Babesia spp. In Domestic Animals in Sudan (North Kordofan and Kassala States)	Springer 2020
359	Defoliation Dynamics in Kikuyugrass Pastures Subjected to Intensities of Defoliation	Medeiros-Neto 2020
360	Evaluation of Greek Cattle Carcass Characteristics (Carcass Weight and Age of Slaughter) Based on SEUROP Classification System	Nikolaou 2020
361	Zoonotic Diseases: Etiology, Impact, and Control	Rahman 2020
362	Effect of flaxseed (Linum usitatissimum) and soybean (Glycine max) oils in Egyptian lactating buffalo and cow diets on the milk and soft cheese quality	Hassan 2020
363	Black oat grown with common vetch improves the chemical composition and degradability rate of forage	Pereira 2020
364	Research on a Low-Cost, Open-Source, and Remote Monitoring Data Collector to Predict Livestock's Habits Based on Location and Auditory Information: A Case Study from Vietnam	Haquangthinhngo 2020
365	Spatial and Temporal Distribution of Cattle Dung and Nutrient Cycling in Integrated Crop–Livestock Systems	Carpinelli 2020
366	Grazing behavior and production characteristics among cows differing in residual feed intake while grazing late season Idaho rangeland	Sprinkle 2020
367	Effect of essential oils on cattle gastrointestinal nematodes assessed by egg hatch, larval migration and mortality testing	Saha 2020
368	Extensive literature search and selection for relevance of studies related to the chemistry and toxicity of glycoalkaloids and quinolizidine alkaloids in food and feed – Final Report	Lanková 2019
369	Mineral-Vitamin Combining Versus Herbal Supplementationto Enhance Performance Ongole Crossbred Bull	Pamungkas 2019
370	Risk factors affecting dairy cattle protective grouping behavior, commonly known as bunching, against Stomoxys calcitrans (L.) On California dairies	El Ashmawy 2019
371	Ingestive behaviour of steers grazing Brachiaria brizantha cultivar Marandu and in feedlot in Brazil	Oliveira 2019
372	Accurate 3D shape recovery of live cattle with three depth cameras	Ruchay 2019
373	Camel milk production and marketing: Pastoral areas of Afar, Ethiopia	Gebremichael 2019
374	Is summer food intake a limiting factor for boreal browsers? Diet, temperature, and reproduction as drivers of consumption in female moose	Shively 2019

375	Management factors affecting adrenal glucocorticoid activity of tourist camp elephants in Thailand and implications for elephant welfare	Bansiddhi 2019
376	Differential role of r-met-hu G-CSF on male reproductive function and development in prepubertal domestic mammals	Aponte 2019
377	Ticks and accompanying pathogens of domestic and wild animals of Kerala, South India	Murikolinimisha 2019
378	Prenatal maternal stress effects on the development of primate social behavior	Schülke 2019
379	Proceedings of the 9th Workshop on Modelling Nutrient Digestion and Utilization in Farm Animals (MODNUT)	
380	Prediction models, assessment methodologies and biotechnological tools to quantify heat stress response in ruminant livestock	Rashamol 2019
381	Impact of weaning age on rumen development in artificially reared lambs	Carballo 2019
382	Improved early postnatal nutrition and its effect on histomorphological parameters in the testes of Sanjabi ram lambs	Moghaddam 2019
383	How many pigs within a group need to be sick to lead to a diagnostic change in the group's behavior? 1	Miller 2019
384	Linseed oil and heated linseed grain supplements have different effects on rumen bacterial community structures and fatty acid profiles in cashmere kids 1	Wang 2019
385	Effect of tree foliage supplementation of tropical grass diet on in vitro digestibility and fermentation, microbial biomass synthesis and enteric methane production in ruminants	Albores-Moreno 2019
386	Linear correlations between feed intake and ingestive behavior of feedlot lambs	Figueiredo 2019
387	Proceedings of the British Society of Animal Science	
388	Biodiscrimination of α-tocopherol stereoisomers in plasma and tissues of lambs fed different proportions of all-rac-α-tocopheryl acetate and RRR-α-tocopheryl acetate	Lashkari 2019
389	Heterochrony of puberty in the European badger (Meles meles) can be explained by growth rate and group-size: Evidence for two endocrinological phenotypes	Sugianto 2019
390	Development of a behavioural welfare assessment tool for routine use with captive elephants	Yon 2019
391	The role of roughage provision on the absorption and disposition of the mycotoxin deoxynivalenol and its acetylated derivatives in calves: from field observations to toxicokinetics	Valgaeren 2019
392	Trichothecenes in Cereal Grains - An Update	Foroud 2019
393	The importance of hoof health in dairy production	Krpálková 2019

394	South Brazilian farmers' perceptions concerning sheep tail docking	Stamm 2019
395	Herbage intake by cattle in kikuyugrass pastures under intermittent stocking method	Schmitt 2019
396	Successes and challenges of the One Health approach in Kenya over the last decade	Munyua 2019
397	Effect of supplementation with n-3 polyunsaturated fatty acids and/or β-glucans on performance, feeding behaviour and immune status of Holstein Friesian bull calves during the pre- and post-weaning periods	Mcdonnell 2019
398	Performance and ingestive behavior of steers on integrated system using legume and/or energy supplementation	Lisbinski 2019
399	Growth performance and rumen development in Malabari kids reared under different production systems	Prasad 2019
400	Time to move beef cattle to a new paddock: forage quality and grazing behaviour	Deoliveira 2018
401	Incorporating natural behavior in housing design and management of lactating sows	Devillers 2018
402	Effect of exogenous butyrate on the gastrointestinal tract of sheep. I. Structure and function of the rumen, omasum, and abomasum	Górka 2018
403	Multiple Feedbacks Contribute to a Centennial Legacy of Reindeer on Tundra Vegetation	Egelkraut 2018
404	TECHNICAL NOTE: Development of a pressure sensor-based system for measuring rumination time in pre-weaned dairy calves	Eslamizad 2018
405	A Review on Organic Farming as a Potential Sector of Agripreneurship Development among the Tribal Youth of India	Dash 2018
406	Preliminary Study to Determinate the Effect of the Rearing Managements Applied during Heifers' Whole Life on Carcass and Flank Steak Quality	Soulat 2018
407	Preferences of Pet Owners in Regard to the Use of Insecticides for Flea Control	Peribáñez 2018
408	Performance-enhancing technologies for steers grazing tall fescue pastures with varying levels of toxicity 1	Diaz 2018
409	Effects of grazing management in brachiaria grass-forage peanut pastures on canopy structure and forage intake	Gomes 2018
410	Proceedings of the 10th International Symposium on the Nutrition of Herbivores	
411	Briefly	
412	Risk of introduction of lumpy skin disease in France by the import of vectors in animal trucks	Saegerman 2018
413	A retrospective pathology study of two Neotropical deer species (1995-2015), Brazil: Marsh deer (Blastocerus dichotomus) and brown brocket deer (Mazama gouazoubira)	Navas-Suárez 2018

414	Behaviour and browse species selectivity of heifers grazing in a temperate silvopastoral system	Vandermeulen 2018
415	Creep Feeding Supplemented with Roughages Improve Rumen Morphology in Pre-Weaning Goat Kids	Naynainghtoo 2018
416	Effect of Different Fat Sources and Vitamin E Levels on Growth Performance, Carcass Characteristics, and Meat Quality of Pigs Grown to 150 Kg	Wang 2018
417	The cultivable autochthonous microbiota of the critically endangered Northern bald ibis (Geronticus eremita)	Spergser 2018
418	Grazing behaviour of dairy cows and body condition score associated with sward characteristics of four pasture types	Castelán-ortega 2018
419	Heat stress and effect of shade materials on hormonal and behavior response of dairy cattle: a review	Kamal 2018
420	Lumpy skin disease II. Data collection and analysis	European Food Safety Authority (EFSA)
421	The impact of early life nutrition and housing on growth and reproduction in dairy cattle	Curtis 2018
422	"Naturalness" and Its Relation to Animal Welfare from an Ethological Perspective	Gygax 2018
423	Keeping the Country Clean: Animal Diseases, Bacteriology, and the Foundations of Biosecurity in New Zealand, 1890-1910	Ford 2018
424	Sward structure and short-term herbage intake in Arachis pintoi cv. Belmonte subjected to varying intensities of grazing	Silva 2018
425	Correlation between production performance and feeding behavior of steers on pasture during the rainy-dry transition period	Brandão 2018
426	Ingestive behaviour and forage intake responses of young and mature steers to the vertical differentiation of sugarcane in pen and grazing studies	Benvenutti 2017
427	Technical note: Instantaneous sampling intervals validated from continuous video observation for behavioral recording of feedlot lambs	Pullin 2017
428	Maternal nutrition during the first 50 days of gestation alters bovine fetal hepatic metabolic transcriptome	Crouse 2017
429	High-resolution palynology reveals the land use history of a Sami renvall in northern Sweden	Kamerling 2017
430	The microbiota-gut-brain axis as a key regulator of neural function and the stress response: Implications for human and animal health 1,2	Wiley 2017
431	Abstracts	
432	Behaviour of horses and cattle at two stocking densities in a coastal salt marsh	Nolte 2017

433	Rewilding-inspired transhumance for the restoration of semiarid silvopastoral systems in Chile	Root-bernstein 2017
434	Herbage intake and milk yield in Comisana ewes as effect of 4 vs 7 h of grazing during late lactation	Valenti 2017
435	Cow behavior recognition based on image analysis and activities	Jingqiu 2017
436	Live animal assessments of rump fat and muscle score in Angus cows and steers using 3-dimensional imaging	Mcphee 2017
437	A novel methodology to assess land-based food self-reliance in the Southwest British Columbia bioregion	Dorward 2017
438	On a Failed Defense of Factory Farming	Puryear 2017
439	Effects of a simulated wolf encounter on brain and blood biomarkers of stress-related psychological disorders in beef cows with or without previous exposure to wolves	Cooke 2017
440	The effect of excluding juveniles on apparent adult olive baboons (Papio anubis) social networks	Fedurek 2017
441	Comparison of culture and a multiplex probe PCR for identifying Mycoplasma species in bovine milk, semen and swab samples	Parker 2017
442	Cyanotoxins: producing organisms, occurrence, toxicity, mechanism of action and human health toxicological risk evaluation	Buratti 2017
443	Rumen fluke (Calicophoron daubneyi) on Welsh farms: prevalence, risk factors and observations on co-infection with Fasciola hepatica	Jones 2017
444	Data collection for risk assessments on animal health (Acronym: DACRAH): Final Report	Dórea 2017
445	Tag SNP selection for prediction of tick resistance in Brazilian Braford and Hereford cattle breeds using Bayesian methods	Sollero 2017
446	Analysis of cortisol in dog hair - a potential biomarker of chronic stress: a review	Mesarcova 2017
447	The Biology and Ecology of Cat Fleas and Advancements in Their Pest Management: A Review	Rust 2017
448	Efficiency of the use of ryegrass by heifers in response to the receival of supplement	Ribeiro 2017
449	The animal trypanosomiases and their chemotherapy: a review	Giordani 2016
450	Supplementation with Ca salts of soybean oil interacts with concentrate level in grazing dairy cows: milk production and milk composition	Macedo 2016
451	Cattle management practices and milk production on mixed smallholder organic pineapple farms in Central Uganda	Nalubwama 2016
452	Predicting forage intake in extensive grazing systems	Galyean 2016

453	International ecohealth One Health Congress 2016	
454	Utility of an online learning module for teaching disbudding in dairy calves, including cornual nerve block application	Winder 2016
455	WS Effects of protein concentration and degradability on performance and carcass characteristics of finishing heifers receiving 0 or 400 mg ractopamine hydrochloride	Samuelson 2016
456	WS Grazing behavior and production characteristics among cows differing in residual feed intake while grazing late season Idaho rangeland	Sprinkle 2016
457	WS Evaluation of Eragrostis tef (Zucc.) As a forage option for grazing beef cattle in the Southern High Plains	Sugg 2016
458	WS Salivary cortisol concentrations affect rumen microbial fermentation and nutrient digestibility in vitro	Samuelson 2016
459	New Publications *	Anonymous 2016
460	Biologically Informed Individual-Based Network Model for Rift Valley Fever in the US and Evaluation of Mitigation Strategies	Scoglio 2016
461	Silica-Triggered Autoimmunity in Lupus-Prone Mice Blocked by Docosahexaenoic Acid Consumption	Bates 2016
462	Housing and Social Environments of African (Loxodonta africana) and Asian (Elephas maximus) Elephants in North American Zoos	Meehan 2016
463	Effect of continuous female exposure on behavioral repertoire and stereotypical behaviors in restrained male dromedary camels during the onset of the breeding season	Fatnassi 2016
464	Seeing the Animal: On the Ethical Implications of De-animalization in Intensive Animal Production Systems	Harfeld 2016
465	In Vivo Pharmacokinetics/Pharmacodynamics of Cefquinome in an Experimental Mouse Model of Staphylococcus Aureus Mastitis following Intramammary Infusion	Yang 2016
466	Use of Extended Characteristics of Locomotion and Feeding Behavior for Automated Identification of Lame Dairy Cows	Beer 2016
467	Alpha proteobacteria of genus Anaplasma (Rickettsiales: Anaplasmataceae): Epidemiology and characteristics of Anaplasma species related to veterinary and public health importance	ATIF 2016
468	Epidemiological Aspects of Bovine Tick Infestation in the River Ravi Region, Lahore	Sadaqatali 2016
469	Summaries	
470	The Nutritional Balancing Act of a Large Herbivore: An Experiment with Captive Moose (Alces alces L)	Felton 2016
471	The Ghastly Kitchen	Guerrini 2016

472	Nellore cattle (Bos indicus) and ticks within the Brazilian Pantanal: ecological relationships	Ramos 2016
473	Behaviors associated with cows more prone to produce milk with reduced stability to ethanol test due to feeding restriction	Stumpf 2016
474	Sward structure, light interception and herbage accumulation in forage peanut cv. Belmonte subjected to strategies of intermittent grazing management	Brunetti 2016
475	Feeding behavior of F1 Holstein x Zebu lactating cows fed increasing levels of banana peel	Pimentel 2016
476	Patterns of use of time by heifers with or without supplementation at different phenological stages of winter grasses	Deoliveira Sichonany 2016
477	A review on the use of sensors to monitor cattle jaw movements and behavior when grazing	Andriamandroso 2016
478	Modelling the potential benefits of different strategies to control infection with Trypanosoma evansi in camels in Somaliland	Salah 2016
479	The effects of concentrate supplementation on growth performance and behavioral activities of cattle grazed on natural pasture	Selemani 2016
480	The Role of Critical Reflexive Analysis in a Service-Learning Course in Agricultural Communication	Borron 2015
481	Gene cloning, expression, and characterization of the Bacillus amyloliquefaciens PS35 lipase	Kanmani 2015
482	Large-Scale Purification of r28m: A Bispecific scfv Antibody Targeting Human Melanoma Produced in Transgenic Cattle	Spiesberger 2015
483	Ecophysiology of C4 Forage Grasses--Understanding Plant Growth for Optimising Their Use and Management	Silva 2015
484	Empirical Methods in Animal Ethics	Persson 2015
485	First molecular evidence of the transplacental transmission of Theileria annulata	Sudan 2015
486	Update on oral vaccination of foxes and raccoon dogs against rabies	Animal Health and Welfare (AHAW)
487	The Effect of Gastrointestinal Nematode Infection Level on Grazing Distance from Dung	Seó 2015
488	Zinc Oxide Nanoparticles: Opportunities and Challenges in Veterinary Sciences	R 2015
489	Male camel behavior and breeding management strategies: How to handle a camel bull during the breeding season?	Padalino 2015
490	Factors influencing the use of willow and birch by moose in winter	Rea 2015
491	Summary List	
492	Phenotypic characteristics and trypanosome prevalence of Mursi cattle breed in the Bodi and	Terefe 2015

	Mursi districts of South Omo Zone, southwest Ethiopia	
493	Coupling models of cattle and farms with models of badgers for predicting the dynamics of bovine tuberculosis (TB)	Moustakas 2015
494	Food intake rates of herbivorous mammals and birds and the influence of body mass	Steuer 2015
495	Laparoscopic abomasal cannulation in sheep	Zhang 2015
496	Ingestive behavior of crossbred heifers in four seasons related to the structure of stargrass pasture	Campana 2015
497	Environmental Drivers of Culicoides Phenology: How Important Is Species-Specific Variation When Determining Disease Policy?	Searle 2014
498	Mixed Stocking by Cattle and Goats for Blackberry Control in Rhizoma Peanut-Grass Pastures	Krueger 2014
499	A "Bovine Glamour Girl": Borden Milk, Elsie the Cow, and the Convergence of Technology, Animals, and Gender at the 1939 New York World's Fair	Hajdik 2014
500	Sequence Analysis and Molecular Characterization of Clonorchis sinensis Hexokinase, an Unusual Trimeric 50-kda Glucose-6-Phosphate-Sensitive Allosteric Enzyme	Chen 2014
501	Identification of Unprecedented Anticancer Properties of High Molecular Weight Biomacromolecular Complex Containing Bovine Lactoferrin (HMW-blf)	Ebrahim 2014
502	Toxicological Evaluation of Lactase Derived from Recombinant Pichia pastoris	Zou 2014
503	Effects of seasonal and climate variations on calves' thermal comfort and behaviour	Tripon 2014
504	A Retrospective Study on the Epidemiology of Anthrax, Foot and Mouth Disease, Haemorrhagic Septicaemia, Peste des Petits Ruminants and Rabies in Bangladesh, 2010-2012	Mondal 2014
505	Attenuated sensing of SHH by Ptch1 underlies evolution of bovine limbs	Lopez-Rios 2014
506	Does Crop Determine Culture?	Hahn 2014
507	Schmallenberg virus: State of Art	
508	Spatial Arrangement of Forages Affects Grazing Behavior of Beef Heifers Continuously Stocked at Low Stocking Rate	Scaglia 2014
509	Update on EFSA's activities on Emerging Risks 2012	
510	Abstracts of Meeting	Anonymous 2014
511	Successful vaccines for naturally occurring protozoal diseases of animals should guide human vaccine research. A review of protozoal vaccines and their designs	Mcallister 2014

512	Refusing to "Push the Cows": The Rise of Organic Dairying in the Northeast and Midwest in the 1970s-1980s	Saucier 2014
513	Neospora caninum Calcium-Dependent Protein Kinase 1 Is an Effective Drug Target for Neosporosis Therapy	Ojo 2014
514	Triple Immunoglobulin Gene Knockout Transchromosomic Cattle: Bovine Lambda Cluster Deletion and Its Effect on Fully Human Polyclonal Antibody Production	Matsushita 2014
515	The European Union Summary Report on Trends and Sources of Zoonoses, Zoonotic Agents and Food-borne Outbreaks in 2012	
516	Could Dromedary Camels Develop Stereotypy? The First Description of Stereotypical Behaviour in Housed Male Dromedary Camels and How It Is Affected by Different Management Systems	Padalino 2014
517	Chopped or Long Roughage: What Do Calves Prefer? Using Cross Point Analysis of Double Demand Functions	Webb 2014
518	Reduction in accuracy of genomic prediction for ordered categorical data compared to continuous observations	Kizilkaya 2014
519	In Vitro Evaluation of Ethanolic Extracts of Ageratum conyzoides and Artemisia absinthium against Cattle Tick, Rhipicephalus microplus	Parveen 2014
520	Effects of Chicory/Perennial Ryegrass Swards Compared with Perennial Ryegrass Swards on the Performance and Carcass Quality of Grazing Beef Steers	Marley 2014
521	Transplacental Transmission of Bluetongue Virus Serotype 1 and Serotype 8 in Sheep: Virological and Pathological Findings	Van der Sluijs 2013
522	Public Health Implications of Animals in Retail Food Outlets	Dyjack 2013
523	Modelling Parasite Transmission in a Grazing System: The Importance of Host Behaviour and Immunity	Fox 2013
524	Effect of feeding goats with distilled and non-distilled thyme leaves (Thymus zygis subp. Gracilis) on milk and cheese properties	Boutoial 2013
525	Physiological Level Production of Antigen-Specific Human Immunoglobulin in Cloned Transchromosomic Cattle	Sano 2013
526	Grazing behaviour, herbage intake and animal performance of beef cattle heifers on marandu palisade grass subjected to intensities of continuous stocking management	Dasilva 2013
527	Posters: T2 NUTRITION THROUGH LIFE COURSE	Anonymous 2013
528	How Much Can Diptera-Borne Viruses Persist over Unfavourable Seasons?	Charron 2013
529	Performance of Beef Cattle Creep Fed Concentrate or Creep Grazed on Warm-Season Legumes	Foster 2013

530	Sex-Age Related Rumination Behavior of Père David's Deer under Constraints of Feeding Habitat and Rainfall	Li 2013
531	Current Approaches to the Determination of Feed Intake and Digestibility in Ruminant Animals - A Review	Kubkomawa 2013
532	Abstracts	Anonymous 2013
533	Telos and the Ethics of Animal Farming	Harfeld 2013
534	The European Union Summary Report on Trends and Sources of Zoonoses, Zoonotic Agents and Food -borne Outbreaks in 2011	
535	Jaguar and puma attacks on livestock in Costa Rica	Amit 2013
536	Vocalization and behavior of Holstein cows and calves after partial and complete separation	Rhim 2013
537	QTL fine mapping with Bayes C(π): a simulation study	Vandenberg 2013
538	"Money talks, bullshit walks" interrogating notions of consumption and survival sex among young women engaging in transactional sex in post-apartheid South Africa: a qualitative enquiry	Zembe 2013
539	Distribution of Artificial Radionuclides in Abandoned Cattle in the Evacuation Zone of the Fukushima Daiichi Nuclear Power Plant	Fukuda 2013
540	Structural Insights into the Dual Strategy of Recognition by Peptidoglycan Recognition Protein, PGRP-S: Structure of the Ternary Complex of PGRP-S with Lipopolysaccharide and Stearic Acid	Sharma 2013
541	Reports on toxicokinetics, toxicity and allergenicity data on substances to be evaluated as acceptable previous cargoes for edible fats and oils	Bassan 2012
542	Specification of data collection on animal diseases to increase the preparedness of the AHAW panel to answer future mandates	Bellet 2012
543	Grazing Behavior of Steers on Different Annual Ryegrass and White Clover Forage Systems	Solomon 2012
544	Present and Potential Future Distribution of Common Vampire Bats in the Americas and the Associated Risk to Cattle	Lee 2012
545	Innate immune responses and health of individually reared Holstein calves after placement into transition-pens 23 d after weaning	Hulbert 2012
546	Scientific report updating the EFSA opinions on the welfare of broilers and broiler breeders	Dejong 2012
547	Systematic literature review on the occurrence of ticks and tick-borne pathogens in the EU and Mediterranean Basin	Maioli 2012
548	Submitted summaries	

549	Do brain cancer rates correlate with ambient exposure levels of criteria air pollutants or hazardous air pollutants (haps)?	Valberg 2012
550	Potential of groundwater contamination by polybrominated diphenyl ethers (pbdes) in a sensitive bedrock aquifer (Canada)	Levison 2012
551	Inventory of available data and data sources and proposal for data collection on vector-borne zoonoses in animals	Mannelli 2012
552	Prevalence of cattle tick infestation in three districts of the punjab, pakistan	Atif 2012
553	Bluetongue virus serotype 1 outbreak in the Basque Country (Northern Spain) 2007-2008. Data support a primary vector windborne transport	García-Lastra 2012
554	Intensive Cattle Grazing Affects Pasture Litter-Fall: An Unrecognized Nitrous Oxide Source	Pal 2012
555	Steer Performance, Intake, Digesta Kinetics, and Pasture Productivity of Flaccidgrass at Each of Three Forage Masses	Burns 2012
556	Research Priorities for Zoonoses and Marginalized Infections: Technical report of the TDR Disease Reference Group on Zoonoses and Marginalized Infectious Diseases of Poverty	Anonymous 2012
557	New records and ecological remarks regarding the tribe Stomoxyini (Diptera: Muscidae) from Israel	Muller 2011
558	Stocking Strategies as Related to Animal and Pasture Productivity of Endophyte-Free Tall Fescue	Burns 2011
559	Scientific Opinion on Pyrrolizidine alkaloids in food and feed	EFSA
560	Past, present and future of pastoralism in Greece	Hadjigeorgiou 2011
561	Scientific Opinion on the public health hazards to be covered by inspection of meat (swine)	EFSA
562	Proteolysis, texture and colour of a raw goat milk cheese throughout the maturation	Delgado 2011
563	Theatre Presentations	
564	Poster Presentations	
565	The effects of feeding fresh forage and silage on some nutritional attributes of beef: an overview	Kalac 2011
566	Tall Fescue Forage Mass and Canopy Characteristics on Steer Ingestive Behavior and Performance	Burns 2011
567	Grazing Behavior and Diet Preference of Beef Steers Grazing Adjacent Monocultures of Tall Fescue and Alfalfa: II. The Role of Novelty	Boland 2011

568	Preparatory work for the future development of animal based measures for assessing the welfare of pig - Report 2: Preparatory work for the future development of animal based measures for assessing the welfare of weaned, growing and fattening pigs including aspects related to space allowance, floor types, tail biting and need for tail docking	Spoolder 2011
569	A review of hemorrhagic septicemia in cattle and buffalo	Shivachandra 2011
570	Potential impact of diseases transmissible by sperm on the establishment of Iberian ibex (Capra pyrenaica) genome resource banks	Santiago-moreno 2011
571	Interrelationships among Forage Nutritive Value and Quantity and Individual Animal Performance	Sollenberger 2011
572	Fine-scale spatial distribution of herbage mass, herbage consumption and fecal deposition by cattle in a pasture under intensive rotational grazing	Hirata 2011
573	1st International One Health Congress Abstracts	Anonymous 2011
574	Behaviour of lame cows: a review	Olechnowicz 2011
575	Bluetongue: a review	Sperlova 2011
576	An Integrated Approach to Modeling Grazing Pressure in Pastoral Systems: The Case of the Logone Floodplain (Cameroon)	Moritz 2010
577	Animal welfare risk assessment guidelines on housing and management (EFSA Housing Risk)	Wageningen UR Livestock Research 2010
578	Plant species selection by free-ranging cattle in southern Bolivian tropical montane forests	Marquardt 2010
579	Defining Environment Risk Assessment Criteria for Genetically Modified Insects to be placed on the EU Market	Benedict 2010
580	Embryo transfer, a useful technique to be applied in small community farms?	Alarcón 2010
581	Bovine tropical theileriosis in a neonate calf	Godara 2010
582	"Our market is our community": women farmers and civic agriculture in Pennsylvania, USA	Trauger 2010
583	Comparative grazing behavior of lactating Holstein-Friesian, Jersey, and Jersey x Holstein-Friesian dairy cows and its association with intake capacity and production efficiency	Prendiville 2010
584	The Most Likely Time and Place of Introduction of BTV8 into Belgian Ruminants	Saegerman 2010
585	Intake and Digestibility of Improved Selections of Tall Fescue and Orchardgrass Hays	Burns 2010
586	Duration of weaning, starter intake, and weight gain of dairy calves fed large amounts of milk	Sweeney 2010
587	Continuous lactation effects on mammary remodeling during late gestation and lactation in dairy goats	Safayi 2010

588	Space use, habitat selection and activity patterns of female Sardinian mouflon (Ovis orientalis musimon) during the lambing season	Ciuti 2009
589	Effect of daily movement of dairy cattle to fresh grass in morning or afternoon on intake, grazing behaviour, rumen fermentation and milk production	Abrahamse 2009
590	Scientific Review on Crimean-Congo Hemorrhagic Fever	
591	Exposure of reptiles to plant protection products	Fryday 2009
592	Compared toxicity of chemicals to reptiles and other vertebrates	Fryday 2009
593	Scientific review on ticks and tick-borne diseases	Pascuccia 2009
594	Scientific review on Tuberculosis in wildlife in the EU	Wilson 2009
595	Disease incidence in ranch and pastoral livestock herds around Lake Mburo National Park, in South Western Uganda	Ocaido 2009
596	Physiological adaptation to the humid tropics with special reference to the West African Dwarf (WAD) goat	Daramola 2009
597	Mycotoxins in animal and human patients	Coppock 2009
598	Pathogen Inactivation In Cow Manure Compost	Erickson 2009
599	"Not to Intrude": A Danish Perspective on Gender and Class in Nineteenth-Century Dairying	Fink 2009
600	Factors affecting intake by grazing ruminants and related quantification methods: a review	Decruyenaere 2009
601	Scientific Opinion on BSE Risk in Bovine Intestines	
602	Restricting time at pasture: Effects on dairy cow herbage intake, foraging behavior, hunger-related hormones, and metabolite concentration during the first grazing session	Gregorini 2009
603	Invited review: The welfare of dairy cattle-Key concepts and the role of science	Vonkeyserlingk 2009
604	Animal Behavior and Soil Nutrient Redistribution in Continuously Stocked Pensacola Bahiagrass Pastures Managed at Different Intensities	Dubeux 2009
605	Development of a Macrophage Cell Culture Method To Isolate and Enrich Francisella tularensis from Food Matrices for Subsequent Detection by Real-Time PCR	Day 2009
606	The impact of cattle pasturing on groundwater quality in bedrock aquifers having minimal overburden	Levison 2009
607	Effects of rumen fill on short-term ingestive behavior and circulating concentrations of ghrelin, insulin, and glucose of dairy cows foraging vegetative micro-swards	Gregorini 2009
608	Volumen 58 -5, Edicion Especial 58 (1951-2009)	Alarcón 2009

609	Lameness, Activity Time-Budgets, and Estrus Expression in Dairy Cattle	Walker 2008
610	Effects of dietary fibre on behaviour and satiety in pigs	Deleeuw 2008
611	Opinion of the Scientific Panel on Animal Health and Welfare on request from the Commission on bluetongue	EFSA
612	Safety of aluminium from dietary intake,Scientific Opinion of the Panel on Food Additives, Flavourings, Processing Aids and Food Contact Materials (AFC)	EFSA
613	The Roles of Labor and Profitability in Choosing a Grazing Strategy for Beef Production in the U.S. Gulf Coast Region	Gillespie 2008
614	Production, Management and the Environment: Calf, Young Stock and Stress Management	Anonymous 2008
615	Forages and Pastures II	Anonymous 2008
616	Forages and Pastures III	Anonymous 2008
617	Animal Behavior and Well-Being: Methodology	Anonymous 2008
618	ADSA-SAD (Student Affiliate Division) Undergraduate Competition: Original Research	Anonymous 2008
619	Effect of thermal stress on physiological parameters, feed intake and plasma thyroid hormones concentration in Alentejana, Mertolenga, Frisian and Limousine cattle breeds	Pereira 2008
620	The Effect of Concrete Floor Roughness on Bovine Claws Using Finite Element Analysis	Franck 2008
621	An update on the ecological distribution of Ixodid ticks infesting cattle in Rwanda: countrywide cross-sectional survey in the wet and the dry season	Bazarusanga 2007
622	Pasteurella multocida and bovine respiratory disease	Dabo 2007
623	Prevalence and seasonal incidence of nematode parasites and fluke infections of sheep and goats in eastern Ethiopia	Sissay 2007
624	Genetic evaluation of growth of Kenya Boran cattle using random regression models	Wasike 2007
625	Texture of Low-Fat Iranian White Cheese as Influenced by Gum Tragacanth as a Fat Replacer	Rahimi 2007
626	Basic Information for the Development of the Animal Welfare Risk Assessment Guidelines	EFSA
627	Poster Sessions	Anonymous 2007
628	Diets Containing Escherichia coli-Derived Phytase on Young Chickens and Turkeys: Effects on Performance, Metabolizable Energy, Endogenous Secretions, and Intestinal Morphology	Pirgozliev 2007
629	A Time to Gather: Foraging with the Bedouins of Galilee	Rosner 2007

630	Impacts of Spatial Patterns in Pasture on Animal Grazing Behavior, Intake, and Performance	Chapman 2007
631	The Value to Herbivores of Plant Physical and Chemical Diversity in Time and Space	Provenza 2007
632	Interaction of Plant Species Diversity on Grazing Behavior and Performance of Livestock Grazing Temperate Region Pastures	Soder 2007
633	Control of ticks of ruminants, with special emphasis on livestock farming systems in India: present and future possibilities for integrated control--a review	Ghosh 2006
634	Diets During Far-Off and Close-Up Dry Periods Affect Periparturient Metabolism and Lactation in Multiparous Cows1	Dann 2006
635	Absorption and Digestive Tract Metabolism of 2-Hydroxy-4-Methylthiobutanoic Acid in Lambs	Lobley 2006
636	Phosphorus appetite in sheep: Dissociating taste from postingestive effects1,2	Villalba 2006
637	A historical study of the traditional livestock merchants of Alsace	Leopauldana 2006
638	Timing of herbage allocation in strip grazing: Effects on grazing pattern and performance of beef heifers1	Gregorini 2006
639	Effects of Continuous Versus Periodic Milk Availability on Behavior and Performance of Dairy Calves	Magvonkeyserlingk 2006
640	Dopamine Antagonist Alters Serum Cortisol and Prolactin Secretion in Lactating Holstein Cows	Ahmadzadeh 2006
641	Thermal balance of cattle grazing winter range: Model application1	Keren 2006
642	Selective behaviour in cattle grazing pastures of strips of birdsfoot trefoil and red clover. 2. The effects of sward maturity and structure	Poli 2006
643	Selective behaviour in cattle grazing pastures of strips of birdsfoot trefoil and red clover. 1. The effects of relative sward area	Poli 2006
644	The Immunomodulatory Effects of Clonidine, an [alpha]-2-Adrenergic Agonist, in Laying Hens	Cheng 2006
645	Sward Structure of Simple and Complex Mixtures of Temperate Forages	Sanderson 2006
646	The effects of host physiology on the attraction of tsetse (Diptera: Glossinidae) and Stomoxys (Diptera: Muscidae) to cattle	Torr 2006
647	The importance of increased levels of oxytocin induced by naloxone to milk removal in dairy cows	Tancin 2006
648	Assessing the Importance of Natural Behavior for Animal Welfare	Bracke 2006
649	Computer-Controlled Milk Feeding of Group-Housed Calves: The Effect of Milk Allowance and Weaning Type	Jensen 2006

650	The Prevalence of Bovine Hypodermosis in Kars Province, Turkey	Kara 2005
651	Influence of Calcium, ph, and Moisture on Protein Matrix Structure and Functionality in Direct-Acidified Nonfat Mozzarella Cheese	Mcmahon 2005
652	Fecal Prevalence and Diversity of Salmonella Species in Lactating Dairy Cattle in Four States	Callaway 2005
653	Rheology, Microstructure, and Functionality of Low-Fat Iranian White Cheese Made with Different Concentrations of Rennet	Madadlou 2005
654	Prepartum Intake, Postpartum Induction of Ketosis, and Periparturient Disorders Affect the Metabolic Status of Dairy Cows	Dann 2005
655	Red fox removal and roe deer fawn survival-a 14-year study	Jarnemo 2005
656	Evaluating the effectiveness of a Mexican strain of Duddingtonia flagrans as a biological control agent against gastrointestinal nematodes in goat faeces	Ojeda-Robertos 2005
657	Effect of accommodating sucking and nosing on the behaviour of artificially reared piglets	Widowski 2005
658	Vitamins A and E: metabolism, roles and transfer to offspring	Debier 2005
659	Competition for Teats and Feeding Behavior by Group-Housed Dairy Calves	Magvonkeyserlingk 2004
660	Cubicle housing systems for cattle: Comfort of dairy cows depends on cubicle adjustment1	Veissier 2004
661	The global importance of ticks	Jongejan 2004
662	Conditioning cattle to graze broom snakeweed (Gutierrezia sarothrae)	Ralphs 2004
663	Intake Regulation and Grazing Behavior of Dairy Cows Under Continuous Stocking	Taweel 2004
664	Blind man	Robinson 2004
665	Molecular epidemiology of antimicrobial resistance in veterinary medicine: where do we go?	Boerlin 2004
666	Development and Analysis of a Rumen Tissue Sampling Procedure	Lesmeister 2004
667	War on foot and mouth disease in the UK, 2001: Towards a cultural understanding of agriculture	Nerlich 2004
668	Predation by red fox on European roe deer fawns in relation to age, sex, and birth date	Jarnemo 2004
669	The effect of bulk density on bite dimensions of cattle grazing microswards in the field	Casey 2004
670	Combined Use of Chymosin and Protease from Cryphonectria parasitica for Control of Meltability and Firmness of Cheddar Cheese	S-Y 2004
671	Phenotypic and genotypic characterization of Escherichia coli O157 strains isolated from humans, cattle and pigs	Osek 2004

672	Fish: a potential source of bacterial pathogens for human beings	Novotny 2004
673	Forages and Pastures: Forages in Dairy Production	Taweel 2004
674	Abstracts tuesday, july 27, 2004: symposia and oral sessions	Anonymous 2004
675	Abstracts thursday, july 29, 2004: symposia and oral sessions	Anonymous 2004
676	Animal behavior & well being ii	Anonymous 2004
677	Forages and Pastures: Forages in Dairy Production	Anonymous 2004
678	Use of nonergot alkaloid-producing endophytes for alleviating tall fescue toxicosis in stocker cattle1,2	Parish 2003
679	Laterality in bovine behavior in an extensive partially suckled herd and an intensive dairy herd	Phillips 2003
680	Grazing of spotted locoweed (Astragalus lentiginosus) by cattle and horses in Arizona	Pfister 2003
681	Canopy characteristics of continuously stocked limpograss swards grazed to different heights	Newman 2003
682	Nutritional limitations to increased production on pasture-based systems	Kolver 2003
683	In Praise of Mock Food	Roberts 2003
684	Fish story	Moody 2003
685	Effects of roughage source and level on intake by feedlot cattle	Galyean 2003
686	A stitch in time: Addressing the environmental, health, and animal welfare effects of China's expanding meat industry	Tao 2003
687	Production, Management, and the Environment	Anonymous 2003
688	Growth & development	Anonymous 2003
689	Invited review: Production and digestion of supplemented dairy cows on pasture	Bargo 2003
690	Selection, intake and excretion of nutrients by Scottish Highland suckler beef cows and calves, and Brown Swiss dairy cows in contrasting Alpine grazing systems	Berry 2002
691	Feed intake patterns, growth performance, and metabolic and endocrine traits in calves fed unlimited amounts of colostrum and milk by automate, starting in the neonatal period	Hammon 2002
692	Canopy morphology and nutritional quality traits as potential grazing value indicators for Lolium perenne varieties	Gilliland 2002
693	The provision of drinking water to veal calves for welfare purposes	Gottardo 2002
694	Germicidal activities of representatives of five different teat dip classes against three bovine mycoplasma species using a modified excised teat model	Boddie 2002
695	Animal Behavior and Well-Being	Anonymous 2002

696	Grazing behavior of ruminants and daily performance from warm-season grasses	Burns 2002
697	Southeastern pasture-based dairy systems: Housing, posilac, and supplemental silage effects on cow performance	Fike 2002
698	Inhibition of oxytocin release during repeated milking in unfamiliar surroundings: the importance of opioids and adrenal cortex sensitivity	Macuhová 2002
699	Field activities and blood profile of pregnant South African indigenous goats after receiving dihydroxy pyridone-degrading rumen bacteria and grazing Leucaena leucocephala-grass or natural pastures	Akingbade 2002
700	Effect of nitrogen on intake and digestibility of a tropical grass grazed by Creole heifers	Boval 2002
701	Abstracts of Original Communications	
702	Effect of suckling on the release of oxytocin, prolactin, cortisol, gastrin, cholecystokinin, somatostatin and insulin in dairy cows and their calves	Lupoli 2001
703	Influence of pasture sward height and concentrate supplementation on intake, digestibility, and grazing time of lactating beef cows	Gekara 2001
704	Consequences of genetic change in farm animals on food intake and feeding behaviour	Emmans 2001
705	Dairy farm management and farm evolution	Peck 2001
706	Does organic farming face distinctive livestock welfare issues? - a conceptual analysis	Alrøe 2001
707	Health and Welfare in Danish Dairy Cattle in the Transition to Organic Production: Problems, Priorities and Perspectives	Vaarst 2001
708	Total radioactive residues and clenbuterol residues in swine after dietary administration of [14C]clenbuterol for seven days and preslaughter withdrawal periods of zero, three, or seven days	Smith 2000
709	Immunization of cross-bred cattle against Hyalomma anatolicum anatolicum by purified antigens	Das 2000
710	The Good Life of Creatures with Dignity Some Comments on the Swiss Expert Opinion	Frans 2000
711	Cross-bred Cattle Protected against Hyalomma anatolicum anatolicum by Larval Antigens Purified by Immunoaffinity Chromatography	Ghosh 1999
712	Social bonding and aggression in female Roosevelt elk	Weckerly 1999
713	Behavior of steers grazing monocultures and binary mixtures of alfalfa and tall fescue	Seman 1999
714	Effects of individual housing design and size on behavior and stress indicators of special-fed Holstein veal calves	Wilson 1999

715	Splanchnic tissue energy use in ruminants that consume forage-based diets ad libitum	Goetsch 1998
716	Comparisons among Tuli-, Brahman-, and Angus-sired heifers: Intake, digesta kinetics, and grazing behavior	Forbes 1998
717	A spatially explicit model of moose foraging and energetics	Moen 1997
718	Improving control of livestock diseases	Teale 1993
