# Supplementary material for: Understanding non-nutritive oral behaviors in dairy calves (Bos taurus): A systematic review protocol
Source: PLoS One. 2025 Mar 20;20(3):e0319778. doi: 10.1371/journal.pone.0319778 (PMC11925274; doi:10.1371/journal.pone.0319778)
Supplement: S5 Table — (PDF) [file pone.0319778.s005.pdf]

S5 Table. Excluded Articles in Round Two of Screening.

Number	Title	First Author, Year	Exclusion Reason
1	Effect of group size on behavior, health, production, and welfare of veal calves	Abdelfattah, 2013	Veal
2	Factors Affecting the Welfare of Unweaned Dairy Calves Destined for Early Slaughter and Abattoir Animal-Based Indicators Reflecting Their Welfare On-Farm	Boyle, 2021	Review
3	Veal calves' clinical/health status in large groups fed with automatic feeding devices	Brscic, 2009	Not on a dairy farm

4	Milk-sucking in dairy cattle in loose housing in Slovakia	Debreceni, 1999	Adult cattle
5	Motivational and physiological analysis of the causes and consequences of non-nutritive sucking by calves	dePassillé, 1997	Review
6	Sucking motivation and related problems in calves	dePassillé, 2001	Review
7	Validation of 1-0 and instantaneous sampling for quantifying oral behaviors in milk-fed dairy calves	Downey, 2021	No treatment or control group
8	Early life access to hay does not affect later life oral behavior in feed-restricted heifers	Downy, 2023	Adult cattle

9	Genetic parameters for abnormal sucking traits in Austrian Fleckvieh heifers	Fuerst-Waltl, 2010	Questionnaire
10	Limit feeding total mixed rations exacerbates intersucking in year-old dairy heifers	Goeller, 2023	Adult cattle
11	On-farm factors associated with cross-sucking in group-housed organic Simmental dairy calves	Größbacher, 2018	Observational only
12	Dam-associated rearing as animal friendly alternative to artificial rearing in dairy cattle	Hillmann, 2012	Does not examine NNOBs

13	Maternal-behavior of dairy heifers and sucking of their newborn calves in group housing	Illman, 1993	Does not examine NNOBs
14	Comparison of time budget of behaviors between penned and ranged young cattle focused on general and oral behaviors	Ishiwata, 2008	Not on a dairy farm

15	Behavior of dairy-cows kept in extensive (loose housing pasture) or intensive (tie stall) environments .3. Grooming, exploration and abnormal-behavior	Krohn, 1994	Adult cattle
16	Which dairy calves are cross-sucked?	Laukkanen, 2010	Observational only
17	Prevalence and potential influencing factors of non-nutritive oral behaviors of veal calves on commercial farms	Leruste, 2014	Not on a dairy farm

18	Factors Associated with Colostrum Quality, the Failure of Transfer of Passive Immunity, and the Impact on Calf Health in the First Three Weeks of Life	Lichtmannsperger, 2023	Does not examine NNOBs
19	Cross-sucking in group-housed dairy calves before and after weaning off milk	Lidfors, 1993	Observational only
20	Influence of milk feeding methods on the welfare of dairy calves	Lidfors, 2009	Review

21	Changes in suckling behaviour of dairy calves nursed by their dam during the first month post partum	Lidfors, 2010	Observational only
22	Effects of the Individual and Pair Housing of Calves on Long-Term Heifer Production on a UK Commercial Dairy Farm	Mahendran, 2024	Does not examine NNOBs
23	The provision of solid feeds to veal calves: II. Behavior, physiology, and abomasal damage	Mattiello, 2002	Veal
24	Performance and feeding behaviour of calves on ad libitum milk from artificial teats	Michael, 2001	Does not examine NNOBs

25	Milk instead of concentrate feed in calf rearing	Neff, 2019	Dutch
26	Early-Life Dam-Calf Contact and Grazing Experience Influence Post-Weaning Behavior and Herbage Selection of Dairy Calves in the Short Term	Nicolao, 2020	Does not examine NNOBs
27	Validation of a methodology for characterization of rumination, lying, standing, and performing non-nutritive oral behaviors and behavioral patterns in Holstein dairy calves	Peng, 2023	No treatment or control group

28	Nutrient intake, feeding patterns, and abnormal behavior of growing bulls fed different concentrate levels and a single fiber source (corn stover silage)	Rahman, 2019	Not on a dairy farm
29	Relations between oral stereotypies, open-field behavior, and pituitary-adrenal system in growing dairy cattle	Redbo, 1998	No treatment or control group

30	The effect of age when group housed and other management factors on playing and non-nutritive sucking behaviour in dairy calves: a cross-sectional observational study	Reipurth, 2020	Observational only
31	Temporal distribution of sucking behaviour in dairy calves and influence of energy balance	Roth, 2009	Observational only
32	Critical control points (CCP) for the housing and management of calves	Schäffer, 2007	Review

33	Performance and Behavioural Responses of Group Housed Dairy Calves to Two Different Weaning Methods	Scoley, 2019	Does not examine NNOBs
34	Development of tongue-playing in artificially reared calves: effects of offering a dummy-teat, feeding of short cut hay and housing system	Seo, 1998	Not on a dairy farm
35	The Impact of Calf Rearing with Foster Cows on Calf Health, Welfare, and Veal Quality in Dairy Farms	Solarczyk, 2023	Veal

36	Intersucking in dairy heifers during the first two years of life	Špinka, 1992	Observational only
37	Chewing activities and particle size of rumen digesta and feces of precision-fed dairy heifers fed different forage levels with increasing levels of distillers grains	Suarez-Mena, 2013	Does not examine NNOBs
38	Blood glucose level and cross-sucking behaviour by different rearing systems in group housed calves	Ude, 2009	Not on a dairy farm
39	Behaviour of calves at introduction to nurse cows after the colostrum period	Vaarst, 2001	Does not examine NNOBs

40	Reciprocated cross sucking between dairy calves after weaning off milk does not appear to negatively affect udder health or production	Vaughan, 2016	Observational only
41	Alternative weaning strategies to diminish acute distress during weaning and separation from the dam after prolonged suckling	Verwer, 2012	Conference proceeding
42	Development of ruminating behavior in Holstein calves between birth and 30 days of age	Wang, 2022	Observational only

43	Behaviour and welfare of veal calves fed different amounts of solid feed supplemented to a milk replacer ration adjusted for similar growth	Webb, 2012	Not on a dairy farm
44	Barren diets increase wakeful inactivity in calves	Webb, 2017	Veal
45	Effect of stall design on dairy calf transition to voluntary feeding on an automatic milk feeder after introduction to group housing	Wilson, 2018	Does not examine NNOBs
