# Supplementary material for: Understanding non-nutritive oral behaviors in dairy calves (Bos taurus): A systematic review protocol
Source: PLoS One. 2025 Mar 20;20(3):e0319778. doi: 10.1371/journal.pone.0319778 (PMC11925274; doi:10.1371/journal.pone.0319778)
Supplement: S6 Table — (PDF) [file pone.0319778.s006.pdf]

S6 Table. Included Articles.

Number	Title	Theme	Main Author, Year
1	Short communication: Effect of age at group housing on behavior, cortisol, health, and leukocyte differential counts of neonatal bull dairy calves	Other - age	Abdelfattah, 2018
2	Effects of intensified or conventional milk feeding on pre-weaning health and feeding behavior of Holstein female calves around weaning	Milk Feeding	Alimirzaei, 2020
3	Effect of individual versus group rearing on ethological and physiological responses of crossbred calves	Housing	Babu, 2004
4	Comparing weaning methods in dairy calves with different dam contact levels	Weaning	Bertelsen, 2023
5	Effects of twice a day teat bucket feeding compared to twice a day mother suckling on behaviour, health traits and blood immune parameters in dairy calves and immune parameters in cow's milk	Milk Feeding	Bieber, 2022
6	Gradual weaning does not improve performance for calves with low starter intake at the beginning of the weaning process	Weaning	Bittar, 2020
7	Behavioural Responses of Dairy Calves to Cafeteria Feeding vs. Single Feeding	Feeding	Boƒüa, 2009
8	Effect of supplemental water source on performance of calves during milk feeding and their cross-sucking after weaning	Other - water	Broucek, 2019
9	Effect of different forage sources on performance and feeding behavior of Holstein calves	Feeding	Castells, 2012
10	Effects of pair versus individual housing on the behavior and performance of dairy calves	Housing	Chua, 2002
11	Effect of age of calf on suckling behaviour and other behavioural activities of Zebu and crossbred calves during restricted suckling periods	Breed	Das, 2000
12	Evaluation of different liquid diets associated with environmental enrichment in the performance and behaviour of dairy calves	Milk Feeding	DaSilva, 2022
13	What components of milk stimulate sucking in calves?	Milk Feeding	dePassillé, 2006
14	Cross-sucking and gradual weaning of dairy calves	Weaning	dePassillé, 2010

15	Cross-sucking by dairy calves may become a habit or reflect characteristics of individual calves more than milk allowance or weaning	Milk Feeding	dePassillé, 2011
16	DOES DRINKING MILK STIMULATE SUCKING IN YOUNG CALVES	Milk Feeding	dePassillé, 1992
17	Some aspects of milk that elicit non-nutritive sucking in the calf	Milk Feeding	dePassillé, 1997
18	Effects of different feeding methods and space allowance on the growth performance, individual and social behaviors of Holstein calves	Housing	Dong, 2017
19	Hay provision affects 24-h performance of normal and abnormal oral behaviors in individually housed dairy calves	Feeding	Downey, 2022
20	Providing long hay in a novel pipe feeder or a bucket reduces abnormal oral behaviors in milk-fed dairy calves	Feeding	Downey, 2023
21	Breed differences in oral behaviors in feed-restricted dairy heifers	Breed	Downey, 2023
22	Development of human-directed behavior in dairy calves reared individually or in pairs	Housing	Doyle, 2022
23	Social behavior of young dairy calves housed with limited or full social contact with a peer	Housing	Duve, 2012
24	Weaning age affects growth, feed intake, gastrointestinal development, and behavior in Holstein calves fed an elevated plane of nutrition during the preweaning stage	Weaning	Eckert, 2015
25	Increased group size reduces conflicts and strengthens the preference for familiar group mates after regrouping of weaned dairy calves (Bos taurus)	Housing	Færevik, 2007
26	Performance, ruminal changes, behaviour and welfare of growing heifers fed a concentrate diet with or without barley straw	Feeding	Faleiro, 2011
27	Effect of restricted suckling on milk yield, milk composition and udder health in cows and behaviour and weight gain in calves, in dual-purpose cattle in the tropics	Milk Feeding	Fröberg, 2007
28	Behaviour of dairy calves suckling the dam in a barn with automatic milking or being fed milk substitute from an automatic feeder in a group pen	Milk Feeding	Fröberg, 2009
29	Performance of free suckling dairy calves in an automatic milking	Milk Feeding	Fröberg, 2011

	system and their behaviour at weaning		
30	Effects of resistance to milk flow and the provision of hay on nonnutritive sucking by dairy calves	Milk Feeding	Haley, 1998
31	Sucking behaviour of dairy calves fed milk ad libitum by bucket or teat	Milk Feeding	Hammell, 1988
32	Feed intake and oral behaviour of dairy calves housed individually or in groups in warm or cold buildings	Housing	Hepola, 2006
33	Effects of providing water from a bucket or a nipple on the performance and behavior of calves fed ad libitum volumes of acidified milk replacer	Other - water feeding method	Hepola, 2008
34	Effects of hunger level and tube diameter on the feeding behavior of teat-fed dairy calves	Milk Feeding	Herskin, 2010
35	The effect of milk-feeding method and hay provision on the development of feeding behavior and non-nutritive oral behavior of dairy calves	Milk Feeding	Horvath, 2017
36	Evaluating effects of providing hay on behavioral development and performance of group-housed dairy calves	Feeding	Horvath, 2019
37	Effects of access to stationary brushes and chopped hay on behavior and performance of individually housed dairy calves	Feeding	Horvath, 2020
38	Impact of enhanced compared to restricted milk feeding on the behaviour and health of organic dairy calves	Milk Feeding	Ivemeyer, 2022
39	The effect of milk flow rate and milk allowance on feeding related behaviour in dairy calves fed by computer controlled milk feeders	Milk Feeding	Jensen, 2003
40	Computer-Controlled Milk Feeding of Dairy Calves: The Effects of Number of Calves per Feeder and Number of Milk Portions on Use of Feeder and Social Behavior	Housing	Jensen, 2004
41	The effects of milk feeding method and group size on feeding behavior and cross-sucking in group-housed dairy calves	Milk Feeding	Jensen, 2006
42	Effect of supplementation fat during the last 3 weeks of uterine life and the preweaning period on performance, ruminal fermentation, blood metabolites, passive immunity and health of the newborn calf	Other- Feed Supplementation	Jolazadeh, 2019

43	The effect of milk quantity and feeding frequency on calf growth and behaviour	Milk Feeding	Jongman, 2020
44	Effects of amount of milk, milk flow and access to a rubber teal on cross-sucking and non-nutritive sucking in dairy calves	Milk Feeding	Jung, 2001
45	Evaluation of environmental and comfort improvements on affective welfare in heifer calves on smallholder dairy farms	Enrichment	Kimeli, 2021
46	Effect of Feeding Method on Nonnutritive Oral Activities in Holstein Calves	Enrichment	Kopp, 1986
47	Long-term effect of colostrum feeding methods on behaviour in female dairy calves	Milk Feeding	Krohn, 1999
48	Effect of initial time of forage supply on growth and rumen development in preweaning calves	Feeding	Lin, 2018
49	Effect of milkflow rate and presence of a floating nipple on abnormal sucking between dairy calves	Milk Feeding	Loberg, 2001
50	Effects of group size on the behaviour, heart rate, immunity, and growth of Holstein dairy calves	Housing	Lv, 2021
51	Effects of feeding frequency of an elevated plane of milk replacer and calf age on behavior, and glucose and insulin kinetics in male Holstein calves	Milk Feeding	MacPherson, 2019
52	Effects of Individual and Pair Housing of Calves on Short-Term Health and Behaviour on a UK Commercial Dairy Farm	Housing	Mahendran, 2023
53	The effect of group housing on behaviour, growth performance, and health of dairy calves	Housing	Malá, 2023
54	Cross-sucking and other oral behaviours in calves, and their relation to cow suckling and food provision	Milk Feeding	Margerison, 2003
55	Effect of milk feeding level on development of feeding behavior in dairy calves	Milk Feeding	Miller-Cushon, 2013
56	Growth performance, feeding behavior, and selected blood metabolites of Holstein dairy calves fed restricted amounts of milk: No interactions between sources of finely ground grain and forage provision	Feeding	Mirzaei, 2017
57	Performance, nutritional behavior, and metabolic responses of calves supplemented with forage depend on starch fermentability	Feeding	Mojahedi, 2018

58	Effect of physical form of forage on performance, feeding behavior, and digestibility of Holstein calves	Feeding	Montoro, 2013
59	Trace minerals source in calf starters interacts with birth weights to affect growth performance	Other- feed supplementation	Mousavi-Haghshenas, 2022
60	Milk allowance and weaning method affect the use of a computer controlled milk feeder and the development of cross-sucking in dairy calves	Milk Feeding	Nielsen, 2008
61	The effects of teat bar design and weaning method on behavior, intake, and gain of dairy calves	Milk Feeding	Nielsen, 2008
62	Effect of portion size and milk flow on the use of a milk feeder and the development of cross-sucking in dairy calves	Milk Feeding	Nielsen, 2018
63	Housing system may affect behavior and growth performance of Jersey heifer calves	Housing	Pempek, 2016
64	The effect of a furnished individual hutch pre-weaning on calf behavior, response to novelty, and growth	Enrichment	Pempek, 2017
65	Sodium chloride supplements increase the salt appetite and reduce stereotypies in confined cattle	Other- feed supplementation	Phillips, 1999
66	The Effects of Forage Provision and Group Size on the Behavior of Calves	Milk Feeding	Phillips, 2004
67	Effects of grain processing (cracked vs. steam-flaked) and forage sources (alfalfa hay and wheat straw) as a free-choice provision on growth performance, rumen fermentation, blood metabolites, and behavior of dairy calves	Feeding	Rahpeyma, 2024
68	Improved weaning reduces cross-sucking and may improve weight gain in dairy calves	Weaning	Roth, 2008
69	Influence of artificial vs. mother-bonded rearing on sucking behaviour, health and weight gain in calves	Milk Feeding	Roth, 2009
70	THE MOTIVATION OF NONNUTRITIVE SUCKING IN CALVES, BOS-TAURUS	Milk Feeding	Rushen, 1995
71	Milk- and starter-feeding strategies to reduce cross sucking in pair-housed calves in outdoor hutches	Milk Feeding	Salter, 2021
72	Does Pelleted Starter Feed Restriction and Provision of Total Mixed Ration Ad Libitum during Weaning Influence the Behavior of Dairy Calves?	Feeding	Spina, 2024

73	Performance and behaviour of calves reared in groups or individually following an enhanced-growth feeding programme	Housing	Terre, 2006
74	Reducing milk induced cross-sucking of group housed calves by an environmentally enriched post feeding area	Feeding	Ude, 2011
75	The effect of unrestricted milk feeding on the growth and health of Jersey calves	Milk Feeding	Uys, 2011
76	Does nutritive and non-nutritive sucking reduce other oral behaviors and stimulate rest in calves?	Milk Feeding	Veissier, 2002
77	Suckling, weaning, and the development of oral behaviours in dairy calves	Milk Feeding	Veissier, 2013
78	Behavioural indicators of hunger in dairy calves	Milk Feeding	Vieira, 2008
79	Understanding oral stereotypies in calves: alternative strategies, hypothalamic-pituitary-adrenal axis (re)activity and gene by environment interactions	Feeding	Webb, 2017
80	Reduction in cross-sucking in calves by the use of a modified automatic teat feeder	Milk Feeding	Weber, 2001
81	Short communication: Pair housing dairy calves in modified calf hutches	Housing	Whalin, 2018
82	A method of outdoor housing dairy calves in pairs using individual calf hutches	Housing	Wormsbecher, 2017
83	Effects of physical enrichment items and social housing on calves? growth, behaviour and response to novelty	Enrichment	Zhang, 2021
84	Holstein calves' preference for potential physical enrichment items on different presentation schedules	Enrichment	Zhang, 2022
