# Supplementary material for: Understanding non-nutritive oral behaviors in dairy calves (Bos taurus): A systematic review protocol
Source: PLoS One. 2025 Mar 20;20(3):e0319778. doi: 10.1371/journal.pone.0319778 (PMC11925274; doi:10.1371/journal.pone.0319778)
Supplement: S7 Table — (PDF) [file pone.0319778.s007.pdf]

S7 Table. Quality Assessment Signaling Questions from Arrive 10 Guidelines.

Domain	Signaling Question(s)
**Study Design**	Are all experimental and control groups clearly identified?
	Is the experimental unit (e.g., individual animal, group, pair etc.) clearly identified?
**Sample Size**	Is the exact number of experimental units in each group at the start of the study provided? (e.g., in the format 'n=')?
	Is the method by which the sample size was chosen explained?
**Inclusion and Exclusion Criteria**	Are the criteria used for including and excluding animals, experimental units, or data points provided?
	Are any exclusions of animals, experimental units, or data points reported, or is there a statement indicating that there were no exclusions?
**Randomization**	Is the method by which experimental units were allocated to control and treatment groups described?
**Blinding**	Is it clear whether researchers were aware of, or blinded to, the group allocation at any stage of the experiment or data analysis (e.g., behaviour coding), if possible?
**Outcome Measures**	For all experimental outcomes presented, are details provided of exactly what parameter was used?
**Statistical methods**	Is the statistical approach used to analyze each outcome detailed?
	Is there a description of any methods used to assess whether data met statistical assumptions?
**Experimental animals**	Are all species of animals used specified?
	Is the sex of the animals specified?
	Is at least one of age, weight, or developmental stage of animals specified?
**Experiment procedure**	Did observers complete inter-observer reliability testing?
	Was each experimental procedure described in enough detail to allow others to replicate them?
**Results**	Are descriptive statistics for each experimental group provided, with a measure of variability (e.g., mean and SD, or median and range)?
	Is the effect size and confidence interval provided?
